# Systematic review of deep learning image analyses for the diagnosis and monitoring of skin disease

**DOI:** 10.1038/s41746-023-00914-8

**Published:** 2023-09-27

**Authors:** Shern Ping Choy, Byung Jin Kim, Alexandra Paolino, Wei Ren Tan, Sarah Man Lin Lim, Jessica Seo, Sze Ping Tan, Luc Francis, Teresa Tsakok, Michael Simpson, Jonathan N. W. N. Barker, Magnus D. Lynch, Mark S. Corbett, Catherine H. Smith, Satveer K. Mahil

**Affiliations:** 1 St John’s Institute of Dermatology, Guy’s and St Thomas’ NHS Foundation Trust and King’s College London, London, UK; 2 St George’s University Hospitals NHS Foundation Trust, London, UK; 3 Maidstone and Tunbridge Wells NHS Trust, Kent, UK; 4 Imperial College London, London, UK; 5 Barking, Havering and Redbridge University Hospitals NHS Trust, London, UK; 6 Department of Medical and Molecular Genetics, King’s College London, London, UK; 7 Center for Reviews and Dissemination, University of York, York, UK

**Keywords:** Skin manifestations, Diagnosis, Medical imaging, Medical research, Disease prevention

## Abstract

Skin diseases affect one-third of the global population, posing a major healthcare burden. Deep learning may optimise healthcare workflows through processing skin images via neural networks to make predictions. A focus of deep learning research is skin lesion triage to detect cancer, but this may not translate to the wider scope of >2000 other skin diseases. We searched for studies applying deep learning to skin images, excluding benign/malignant lesions (1/1/2000-23/6/2022, PROSPERO CRD42022309935). The primary outcome was accuracy of deep learning algorithms in disease diagnosis or severity assessment. We modified QUADAS-2 for quality assessment. Of 13,857 references identified, 64 were included. The most studied diseases were acne, psoriasis, eczema, rosacea, vitiligo, urticaria. Deep learning algorithms had high specificity and variable sensitivity in diagnosing these conditions. Accuracy of algorithms in diagnosing acne (median 94%, IQR 86–98; *n* = 11), rosacea (94%, 90–97; *n* = 4), eczema (93%, 90–99; *n* = 9) and psoriasis (89%, 78–92; *n* = 8) was high. Accuracy for grading severity was highest for psoriasis (range 93–100%, *n* = 2), eczema (88%, *n* = 1), and acne (67–86%, *n* = 4). However, 59 (92%) studies had high risk-of-bias judgements and 62 (97%) had high-level applicability concerns. Only 12 (19%) reported participant ethnicity/skin type. Twenty-four (37.5%) evaluated the algorithm in an independent dataset, clinical setting or prospectively. These data indicate potential of deep learning image analysis in diagnosing and monitoring common skin diseases. Current research has important methodological/reporting limitations. Real-world, prospectively-acquired image datasets with external validation/testing will advance deep learning beyond the current experimental phase towards clinically-useful tools to mitigate rising health and cost impacts of skin disease.

## Introduction

The digitisation of healthcare, accelerated by the COVID-19 pandemic, has led to an accumulation of ‘big data’, so called not only for its substantial volume but also for its complexity and diversity. The availability of such data and advances in computing capacity provide a unique opportunity to revolutionise healthcare using artificial intelligence (AI)^1^.

Machine learning (ML) is a subfield of AI that uses computational models to perform intelligent predictions based on training datasets without direct human intervention^2^. In recent years, deep learning (DL) has become the most widely used computational approach in the field of ML^3^. DL, inspired by the information processing patterns of the human brain, is based on multi-layered artificial neural networks that learn from big data. Broadly, there are 3 types of datasets used in a DL study: training, validation and testing. A training dataset is used to derive DL models, where the algorithms are ‘fitted’ to perform particular functions. A validation dataset is then used to provide an evaluation of the performance of the model, whilst finetuning its architecture. The test dataset provides the final evaluation of the model^4^.

Interest in medical applications of DL has largely been in the fields of radiology, ophthalmology, pathology, and dermatology^5^. As a visual specialty with large image databases, dermatology has considerable potential to augment disease diagnosis and severity assessment using DL, leading to improved healthcare efficiency and reduced costs. A major focus of DL research (bolstered by expanding big data, image quality, computing capacity and DL techniques) is in assisting clinicians in the diagnosis of skin cancers from images^6–8^. This has achieved encouraging results with a meta-analysis of 70 studies suggesting that the accuracy of computer-aided (including DL-based) diagnosis of melanoma was comparable to that of dermatologists^6^.

Whilst this area of dermatology remains a focus of research, easing the burden of non-lesion dermatological diseases warrants attention owing to their high prevalence, visibility, psychosocial impact, need for long-term treatment and associated costs. An estimated 20–25% of the population is affected by chronic inflammatory skin diseases, the most common of which include eczema and psoriasis^9^. The delayed access to healthcare and unpredictable clinical course of inflammatory skin diseases further adds to the already substantial impact on quality of life and underlines the potential for improved, early diagnosis and close monitoring approaches that leverage DL image analysis.

This systematic review assesses the evidence for using DL image analyses in the diagnosis and severity monitoring of skin diseases, beyond benign and malignant skin lesions.

## Results

### Study screening

**Fig. 1:**
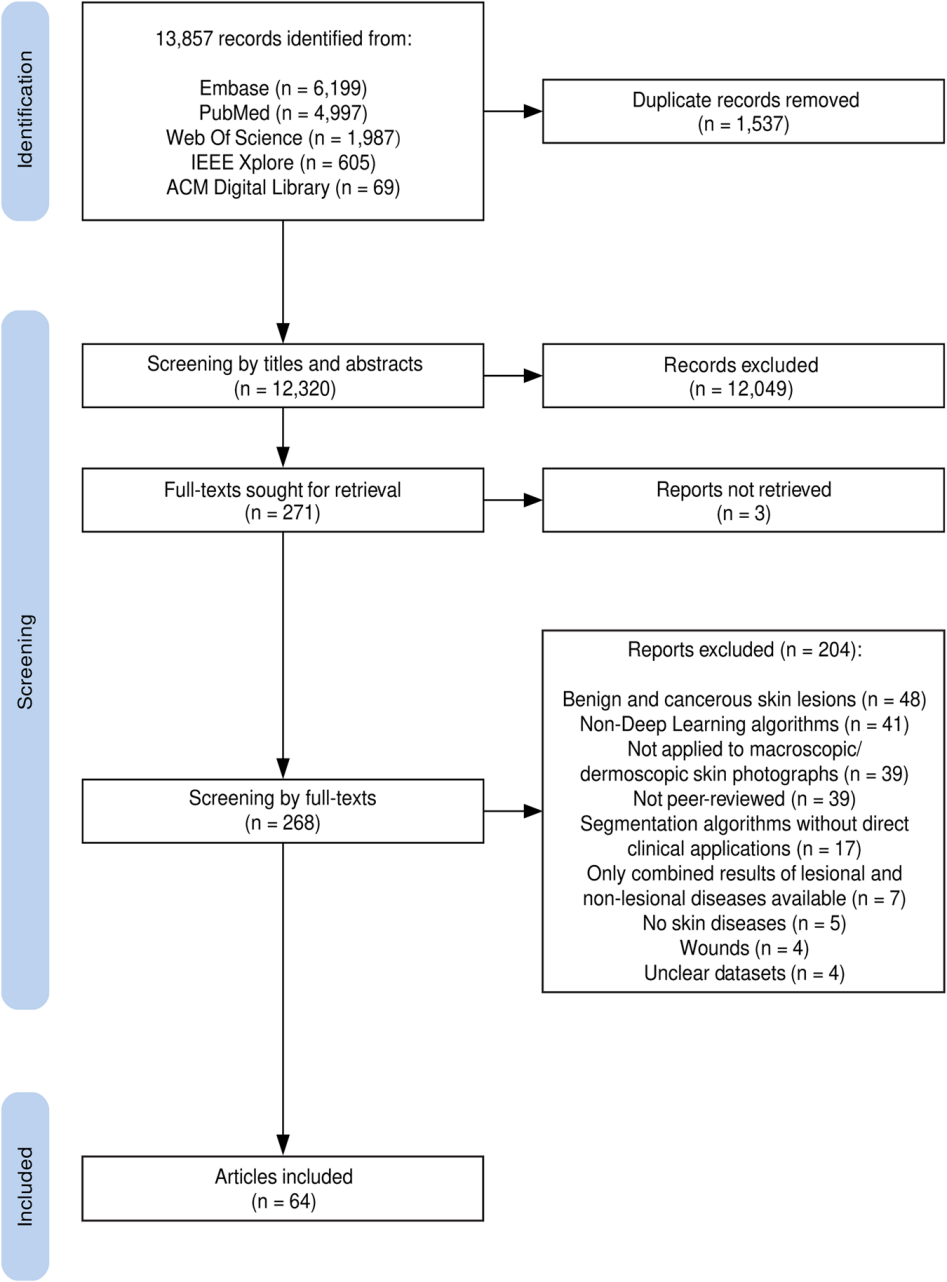
PRISMA flowchart of study records. PRISMA flowchart showing the study selection process.

The searches identified 13,857 references. After removing duplicates, 12,320 titles and abstracts were screened. Subsequent full-text screening of 268 studies identified 64 studies that met the eligibility criteria (Fig. 1 and Supplementary Material 1). No studies prior to 2012 met our eligibility criteria. This is in keeping with the paradigm shift towards the use of DL in ML research in 2012, when AlexNet (a type of DL) was shown to significantly outperform traditional ML image analysis methods^10^.

### Quality assessment

**Fig. 2:**
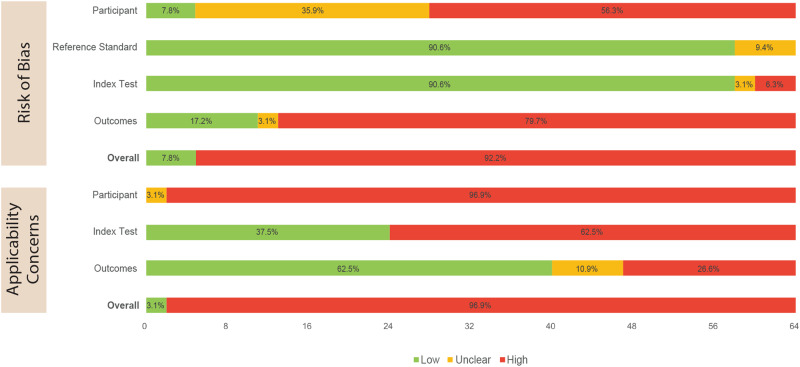
Summary of quality assessment results of all studies (using modified QUADAS-2). Quality assessment of all included studies.

Risk of bias and applicability concerns for all 64 included studies were assessed using our modified QUADAS-2 framework (Supplementary Material 2). 59 studies (92%) had overall high risk of bias judgements, and 62 studies (97%) had overall high level applicability concerns (Fig. 2 and Supplementary Material 3). Of 9 studies that used external datasets to validate or test their DL algorithms, 8 studies (89%) still had an overall high risk of bias and all 9 studies (100%) had overall high level applicability concerns (Supplementary Material 4).

With respect to risk of bias, the participant and outcome domains were more commonly rated as high/unclear (92% and 83%, respectively), in contrast to the reference standard and index test domains (9% and 9%, respectively) (Fig. 2). To determine the reference standard, most studies (61%, *n* = 39) had datasets verified by at least one clinician. With respect to the index test, 91% (*n* = 58) accounted for overfitting, underfitting, and/or optimism when assessing DL algorithm performance against the reference standard.

All 64 studies scored high or unclear in the participant domain of applicability concerns (Fig. 2). All externally validated/tested DL algorithms (*n* = 9) also had high level applicability concerns in this domain (Supplementary Material 4). There was poor reporting of participant characteristics such as Fitzpatrick skin type, age, and gender, as well as poor generalisability of the study settings. 63% and 38% of all studies had high/unclear applicability concerns in the index test and outcome domains, respectively (Fig. 2). This reduced to 0% and 11%, respectively, when considering only externally validated/tested DL algorithms (Supplementary Material 4).

### General study characteristics

Overall, 144 skin diseases were studied. Of these, the most frequently studied diseases were acne (*n* = 30), psoriasis (*n* = 27), eczema (*n* = 22), rosacea (*n* = 12), vitiligo (*n* = 12) and urticaria (*n* = 8) (Tables 1 and 2). The most common skin disease categories were inflammatory, follicular, pigmentary and infectious disorders (Table 3).

**Table 1:** Baseline characteristics of all studies.

Skin disease		Acne	Psoriasis	Eczema	Rosacea	Vitiligo	Urticaria	Other†	Overall
**Number of included studies**		30	27	22	12	12	8	38	64
**Study design N (%)**	Prospective	2 (6.7)	5 (18.5)	3 (13.6)	0 (0.0)	1 (8.3)	0 (0.0)	6 (15.8)	8 (12.5)
	Retrospective	28 (93.3)	22 (81.5)	19 (86.4)	12 (100.0)	11 (91.7)	8 (100.0)	32 (84.2)	56 (87.5)
**Number of images**	Training*	1630 (548–10,975)	1549 (633–7267)	4740 (948–13,235)	12350 (685–20,000)	12350 (1295–30,471)	7621 (330–12,350)	4006 (1038–12,793)	2555 (902–8550)
	Validation*	1430 (894–8275)	1449 (385–3620)	1654 (588–12,350)	12350 (1145–32,755)	1449 (281–20,000)	6900 (1430–28,930)	1116 (274–1672)	1032 (274–2000)
	Testing*	307 (150–1014)	213 (157–594)	323 (184–824)	209 (164–388)	579 (261–2058)	360 (196–2058)	344 (200–1048)	331 (157–922)
	Reported**–Training N (%)	24 (88.9)	24 (100.0)	19 (100.0)	11 (100.0)	9 (100.0)	6 (100.0)	33 (97.1)	56 (93.3)
	Reported**–Validation N (%)	12 (80.0)	13 (92.9)	11 (91.7)	5 (83.3)	7 (100.0)	4 (100.0)	18 (94.7)	31 (91.2)
	Reported**–Testing N (%)	26 (100.0)	20 (100.0)	17 (100.0)	10 (100.0)	9 (100.0)	5 (100.0)	30 (93.8)	50 (96.2)
**Number of participants**	Training*	2269 (903–7045)	1852 (110–7045)	2269 (1583–7469)	2269 (n/a)	7200 (n/a)	9722 (n/a)	2538 (2000–7045)	2000 (416–5860)
	Validation*	3102 (1208–5625)	2157 (100–5625)	2000 (n/a)	1112 (n/a)	3102 (n/a)	5625 (n/a)	2000 (463–5625)	626 (167–3102)
	Testing*	241 (185–340)	241 (145–340)	241 (145–340)	241 (n/a)	340 (n/a)	281 (n/a)	200 (90–398)	185 (90–340)
	Reported**–Training N (%)	6 (22.2)	6 (25.0)	4 (21.1)	2 (18.2)	2 (22.2)	2 (33.3)	7 (20.6)	11 (18.3)
	Reported**–Validation N (%)	4 (26.7)	4 (28.6)	3 (25.0)	2 (33.3)	2 (28.6)	2 (50.0)	5 (26.3)	8 (23.5)
	Reported**–Testing N (%)	4 (15.4)	4 (20.0)	4 (23.5)	2 (20.0)	2 (22.2)	1 (20.0)	7 (21.9)	8 (15.4)
**Ethnicity/Fitzpatrick scale reported N (%)**		8 (26.7)	9 (33.3)	8 (36.4)	3 (25.0)	5 (41.7)	4 (50.0)	8 (21.1)	12 (18.8)
**Type of images N (%)**	Macroscopic images	28 (93.3)	22 (81.5)	19 (86.4)	10 (83.3)	10 (83.3)	7 (87.5)	31 (81.6)	56 (87.5)
	Dermoscopic images	1 (3.3)	4 (14.8)	2 (9.1)	1 (8.3)	0 (0.0)	0 (0.0)	4 (10.5)	5 (7.8)
	Both (macroscopic & dermoscopic)	1 (3.3)	1 (3.7)	2 (4.5)	1 (8.3)	2 (16.7)	1 (12.5)	3 (7.9)	3 (4.7)
**Image source N (%)**	Public database	11 (36.7)	5 (18.5)	6 (27.3)	4 (33.3)	2 (16.7)	3 (37.5)	8 (21.1)	14 (21.9)
	Single-centre hospital/university database	8 (26.7)	11 (40.7)	7 (31.8)	3 (25.0)	3 (25.0)	0 (0.0)	11 (28.9)	23 (35.9)
	Multi-centre hospital/university database	3 (10.0)	5 (18.5)	5 (22.7)	2 (16.7)	3 (25.0)	3 (37.5)	5 (13.2)	7 (10.9)
	Mixed	5 (16.7)	5 (18.5)	3 (13.6)	2 (16.7)	3 (25.0)	2 (25.0)	10 (26.3)	15 (23.4)
	Others	2 (6.7)	0 (0.0)	0 (0.0)	0 (0.0)	0 (0.0)	0 (0.0)	2 (5.3)	2 (3.1)
	Unavailable	1 (3.3)	1 (3.7)	1 (4.5)	1 (8.3)	1 (8.3)	0 (0.0)	2 (5.3)	3 (4.7)
**Function of DL algorithm**	Diagnosis	24 (80.0)	23 (85.2)	21 (95.5)	12 (100.0)	12 (100.0)	8 (100.0)	37 (97.4)	52 (81.3)
	Severity	6 (20.0)	4 (14.8)	1 (4.5)	0 (0.0)	0 (0.0)	0 (0.0)	1 (2.6)	12 (18.8)
**Reference standard**	Dermatologists	13 (43.3)	14 (51.9)	10 (45.5)	4 (33.3)	4 (33.3)	2 (25.0)	13 (34.2)	27 (42.2)
	Non-dermatology clinicians	2 (6.7)	3 (11.1)	1 (4.5)	1 (8.3)	1 (8.3)	1 (12.5)	6 (15.8)	9 (14.1)
	Mixed	4 (13.3)	4 (14.8)	5 (22.7)	1 (8.3)	3 (25.0)	2 (25.0)	7 (18.4)	9 (14.1)
	Other	4 (13.3)	3 (11.1)	2 (9.1)	3 (25.0)	0 (0.0)	1 (12.5)	4 (10.5)	4 (6.3)
	Unavailable	7 (23.3)	3 (11.1)	4 (18.2)	3 (25.0)	4 (33.3)	2 (25.0)	8 (21.1)	15 (23.4)
**Type of study**	Training only	24 (80.0)	23 (85.2)	16 (72.7)	9 (75.0)	6 (50.0)	5 (62.5)	31 (81.6)	54 (84.4)
	Training and external validation	0 (0.0)	0 (0.0)	1 (4.5)	0 (0.0)	0 (0.0)	0 (0.0)	0 (0.0)	1 (1.6)
	Training and external testing	3 (10.0)	1 (3.7)	2 (9.1)	2 (16.7)	3 (25.0)	1 (12.5)	3 (7.9)	5 (7.8)
	External testing only	2 (6.7)	2 (7.4)	2 (9.1)	1 (8.3)	2 (16.7)	1 (12.5)	3 (7.9)	3 (4.7)
	Internal testing only^+^	1 (3.3)	1 (3.7)	1 (4.5)	0 (0.0)	1 (8.3)	1 (12.5)	1 (2.6)	1 (1.6)
**Type of algorithm**^±^	(Deep) CNN	26 (83.9)	25 (89.3)	18 (75.0)	12 (92.3)	11 (84.6)	7 (77.8)	32 (74.4)	55 (79.7)
	Multilayer perceptron	1 (3.2)	0 (0.0)	2 (8.3)	0 (0.0)	0 (0.0)	1 (11.1)	1 (2.3)	2 (2.9)
	Artificial neural network	1 (3.2)	1 (3.6)	2 (8.3)	0 (0.0)	1 (7.7)	0 (0.0)	2 (4.7)	2 (2.9)
	Ensemble	2 (6.5)	1 (3.6)	1 (4.2)	1 (7.7)	1 (7.7)	1 (11.1)	5 (11.6)	6 (8.7)
	Unspecified deep learning	1 (3.2)	1 (3.6)	1 (4.2)	0 (0.0)	0 (0.0)	0 (0.0)	3 (7.0)	4 (5.8)
**Availability of data**	Public data	4 (13.3)	2 (7.4)	4 (18.2)	3 (25.0)	1 (8.3)	1 (12.5)	6 (15.8)	9 (14.1)
	Partially available	3 (10.0)	1 (3.7)	0 (0.0)	0 (0.0)	1 (8.3)	1 (12.5)	2 (5.3)	5 (7.8)
	Available on request	3 (10.0)	4 (14.8)	3 (13.6)	1 (8.3)	0 (0.0)	0 (0.0)	4 (10.5)	6 (9.4)
	Unavailable	20 (66.7)	20 (74.1)	15 (68.2)	8 (66.7)	10 (83.3)	6 (75.0)	26 (68.4)	44 (68.8)
**Availability of DL code**	Public domain	7 (23.3)	5 (18.5)	6 (27.3)	2 (16.7)	2 (16.7)	2 (25.0)	8 (21.1)	13 (20.3)
	Opensource code	3 (10.0)	5 (18.5)	2 (9.1)	1 (8.3)	2 (16.7)	0 (0.0)	7 (18.4)	13 (20.3)
	Unavailable	20 (66.7)	17 (63.0)	14 (63.6)	9 (75.0)	8 (66.7)	6 (75.0)	23 (60.5)	38 (59.4)
**Description of algorithm**	Words and flowchart	20 (66.7)	19 (70.4)	13 (59.1)	7 (58.3)	6 (50.0)	3 (37.5)	28 (73.7)	46 (71.9)
	Words only	4 (13.3)	3 (11.1)	3 (13.6)	3 (25.0)	3 (25.0)	2 (25.0)	4 (10.5)	10 (15.6)
	Flowchart only	0 (0.0)	1 (3.7)	1 (4.5)	0 (0.0)	0 (0.0)	0 (0.0)	1 (2.6)	1 (1.6)
	Unavailable	6 (20.0)	4 (14.8)	5 (22.7)	2 (16.7)	3 (25.0)	3 (37.5)	5 (13.2)	7 (10.9)

The six most commonly studied diseases are presented in individual columns (acne, psoriasis, eczema, rosacea, vitiligo, urticaria). Studies assessing multiple diseases are reported in each of the relevant disease columns. The ‘overall’ values (final column) may not equal the sum of the previous columns (i.e. acne, psoriasis, eczema, rosacea, vitiligo, urticaria, other), as studies of multiple diseases have only been counted once in the ‘overall’ column.

*Median (IQR).

**The percentages (in brackets) are the proportion of total studies performing that particular phase of a deep learning (*DL*) study (i.e. training, validation, testing) that report the number of images/participants. Not all studies completed all three phases, so the denominators may not necessarily sum to the total number of included studies.

^+^1 study (Jain et al. 2021) was labelled as ‘internal testing’ as it did not fit into any of the categories from our modified PROBAST definitions. This study did not develop or validate any new DL algorithms. It tests a previously developed DL algorithm using the same datasets previously used to validate it in a separate study/publication.

^±^Total number of algorithms may not sum to the total number of studies, as some studies used multiple algorithms.

^†^Any disease that cannot be categorised as one of the six most commonly studied diseases.

Deep learning (*DL*), Convolutional neural network (*CNN*).

**Table 2:** Outcomes of deep learning algorithms for the diagnosis of the six most studied diseases.

	Outcome											
	Accuracy (%)		AUC		Sensitivity (%)		Specificity (%)		PPV (%)		NPV (%)	
	All studies	Externally validated/tested studies	All studies	Externally validated/tested studies	All studies	Externally validated/tested studies	All studies	Externally validated/tested studies	All studies	Externally validated/tested studies	All studies	Externally validated/tested studies
**Acne**												
**Median (IQR)**	93.5 (85.7–97.5)	91.9 (n/a)	0.98 (0.93–0.99)	0.98 (n/a)	89.9 (82.2–96.3)	87.0 (n/a)	95.2 (92.9–97.6)	98.2 (n/a)	86.5 (81.3–87.5)	87.2 (n/a)	96.0 (n/a)	98.6 (n/a)
**Range**	79.0–99.7	84.0–99.7	0.89–0.99	0.98	67.0–100.0	84.0–89.9	92.1–100.0	98.2	78.6–100.0	86.9–87.5	93.4–98.6	98.6
**Number of studies**	11	2	4	1	11	2	8	1	10	2	2	1
**Psoriasis**												
**Median (IQR)**	89.1 (78.1–92.0)	73.7 (n/a)	0.93 (0.84–0.98)	0.93 (n/a)	90.0 (73.7–92.0)	75.7 (n/a)	95.4 (90.1–97.1)	96.1 (n/a)	82.4 (60.6–88.6)	72.6 (n/a)	94.8 (n/a)	98.1 (n/a)
**Range**	69.4–98.5	73.7	0.81–0.99	0.93	60.0–95.6	73.7–77.7	88.2–98.8	96.1	60–95.5	62.8–82.4	91.5–98.1	98.1
**Number of studies**	8	1	5	1	10	2	8	1	7	2	2	1
**Eczema**												
**Median (IQR)**	92.6 (89.7–99.4)	95.8	0.93 (0.87–0.99)	0.87 (n/a)	83.8 (70.2–94.6)	73.0 (n/a)	95.8 (92.6–98.8)	97.6 (n/a)	77.1 (61.9–89.7)	81.1 (n/a)	93.2 (n/a)	95.8 (n/a)
**Range**	83.9–99.9	91.7–99.9	0.79–0.99	0.87	54.3–99.6	54.3–91.7	86.6–99.6	97.6	43.0–98.9	67.8–94.3	90.5–95.8	95.8
**Number of studies**	9	2	6	1	13	2	10	1	8	2	2	1
**Rosacea**												
**Median (IQR)**	93.7 (89.6–96.9)	n/a	0.90 (0.87–0.94)	0.91 (n/a)	63.4 (41.7–92.0)	41.7 (n/a)	97.0 (93.9–99.3)	99.8 (n/a)	89.8 (35.7–94.5)	35.7 (n/a)	95.1 (n/a)	99.9 (n/a)
**Range**	87.8–97.9	n/a	0.85–0.97	0.91	0.0–100.0	41.7	91.7–99.8	99.8	0.0–95.0	35.7	90.2–99.9	99.9
**Number of studies**	4	0	4	1	6	1	5	1	**7**	**1**	2	1
**Vitiligo**												
**Median (IQR)**	87.8 (n/a)	100.0 (n/a)	0.98 (n/a)	0.98 (n/a)	90.9 (80.4–95.1)	82.7 (n/a)	88.3 (79.8–97.6)	98.8 (n/a)	90.9 (n/a)	80.1 (n/a)	99.6 (n/a)	99.6 (n/a)
**Range**	85.7–100.0	100.0	0.94–1.00	0.98	72.4–97.2	72.4–92.9	79.4–98.8	98.8	80.1–91.9	80.1	99.6	99.6
**Number of studies**	3	1	3	1	5	2	4	1	3	1	1	1
**Urticaria**												
**Median (IQR)**	80.6 (n/a)	n/a	0.91 (n/a)	0.91 (n/a)	65.8 (n/a)	55.7 (n/a)	99.8 (n/a)	99.8 (n/a)	76.9 (n/a)	75.6 (n/a)	99.5 (n/a)	99.5 (n/a)
**Range**	68.3–92.8	n/a	0.91	0.91	55.7–75.9	55.7	99.7–99.8	99.8	75.6–78.2	75.6	99.5	99.5
**Number of studies**	2	0	1	1	2	1	2	1	2	1	1	1

The six most studied diseases are acne, psoriasis, eczema, rosacea, vitiligo and urticaria. Studies assessing multiple diseases are reported in each of the relevant disease columns. Where studies report multiple outcomes by using variations of DL algorithms or datasets, the best performing results are presented. Outcomes for ‘externally validated/tested studies’ (i.e. where datasets independent from the training dataset are used for validation and/or testing DL algorithms) are presented separately from ‘all studies’, as these studies are presumed to be at a lower risk of overfitting. Interquartile ranges (IQR) are not presented for less than four studies.

Deep learning (*DL*), area under the receiver operating characteristic curve (*AUC*), positive predictive value (*PPV*), negative predictive value (*NPV*), interquartile range (*IQR*).

**Table 3:** Outcomes of deep learning algorithms for the diagnosis of the five main categories of skin disease.

Outcome						
	Accuracy (%)		Sensitivity (%)		Specificity (%)	
	All studies	Externally validated/tested studies	All studies	Externally validated/tested studies	All studies	Externally validated/tested studies
**Inflammatory disorders**						
(psoriasis, eczematous disorders, lichenoid disorders, immunobullous diseases, angioedema, urticaria, discoid lupus erythematous, fixed drug eruption, papulosquamous eruptions)						
** Median (IQR)**	91.6 (80.0–95.8)	82.7 (52.9–99.9)	77.3 (63.3–92.0)	58.1 (48.4–71.6)	97.8 (94.8–99.3)	99.5 (97.6–99.8)
** Range**	35.0–100.0	35.0–100.0	35.3–99.6	35.3–91.7	71.6–100.0	95.7–100.0
** Number of studies**	30	6	47	12	40	10
**Follicular disorders of skin**						
(acne, rosacea, hidradenitis suppurativa)						
** Median (IQR)**	93.0 (86.8–96.7)	84.0 (n/a)	87.4 (67.0–93.9)	86.9 (62.8–91.9)	96.9 (93.0–98.9)	94.4 (n/a)
** Range**	49.3–99.7	49.3–99.7	0.0–100.0	41.7–93.9	91.7–100.0	94.1–94.6
** Number of studies**	16	3	19	4	15	2
**Alopecia**						
** Median (IQR)**	100.0 (n/a)	100.0 (n/a)	84.1 (83.0–94.1)	94.1 (n/a)	99.6 (n/a)	99.3 (n/a)
** Range**	100.0	100.0	82.0–100.0	88.1–100.0	99.3–99.8	99.3
** Number of studies**	2	2	5	2	2	1
**Pigmentary disorders**						
(vitiligo, melasma)						
** Median (IQR)**	87.8 (80.4–99.0)	98.0 (n/a)	86.1 (73.7–91.9)	79.4 (73.7–88.4)	97.4 (80.2–98.8)	98.6 (n/a)
** Range**	75.0–100.0	75.0–100.0	71.9–97.2	72.4–92.9	79.4–98.9	98.5–98.8
** Number of studies**	5	3	8	4	6	2
**Skin infections**						
(viral, bacterial, fungal, parasitic skin infections)						
** Median (IQR)**	87.5 (60.2–94.9)	59.3 (50.0–73.7)	76.9 (63.1–92.5)	70.2 (55.8–80.3)	98.9 (93.2–99.7)	99.1 (97.4–99.8)
** Range**	26.7–100.0	26.7–95.6	26.7–100.0	26.7–96.9	72.7–100.0	72.7–99.9
** Number of studies**	17	7	33	17	25	13

The five skin disease categories are inflammatory disorders, follicular disorders of skin, alopecia, pigmentary disorders and skin infections. Studies assessing multiple diseases are reported under each of the relevant disease categories. Where studies report multiple outcomes by using variations of DL algorithms or datasets, the best performing results are presented. Outcomes for ‘externally validated/tested studies’ (i.e. where datasets independent from the training dataset are used for validation and/or testing DL algorithms) are presented separately from ‘all studies’, as these studies are presumed to be at a lower risk of overfitting.

Deep learning *(DL)*, interquartile range *(IQR)*.

47 of 64 (73%) included studies reported research funding, 6 (9%) did not and 11 (17%) were unclear (Supplementary Material 5). The authors were most frequently affiliated to China (*n* = 20), India (*n* = 9) and the USA (*n* = 5), and private datasets were mostly from Asia (73%, *n* = 35) (Supplementary Material 6).

### Study design

Most studies (88%, *n* = 56) used retrospectively collected data and most (85%, *n* = 55) used the same image dataset for both training and validation/testing (Table 1). Few studies (14%, *n* = 9) used independent external data to validate or test their DL algorithms (Supplementary Material 7). Overall, 24 studies (37.5%) evaluated the algorithm in an independent dataset, a clinical setting or prospectively. No RCTs of DL in skin diseases were found.

DL algorithms were developed predominantly for disease diagnosis (81%, *n* = 52), rather than severity assessment (19%, *n* = 12). Diagnostic DL algorithms were most commonly developed for acne (*n* = 24), psoriasis (*n* = 23) and eczema (*n* = 21) (Table 1). Disease severity DL algorithms were most commonly developed for acne (*n* = 6) and psoriasis (*n* = 4).

### Participants and images

Of those studies performing training (*n* = 60), internal/external validation (*n* = 34) and internal/external testing (*n* = 52) of DL algorithms, the number of participants was reported by 18% (median 2000 participants, IQR 416–5860; *n* = 11), 24% (median 626 participants, IQR 167–3102; *n* = 8) and 15% (median 185 participants, IQR 90–340; *n* = 8), respectively (Table 1). Participant age was reported in 13 (20%) studies and sex was reported in 12 (19%) studies.

In the minority of studies reporting participant ethnicity and/or Fitzpatrick skin type (19%, *n* = 12; Table 1), there was representation across most ethnicities and skin types. Of 10 studies reporting Fitzpatrick skin types, 4 specified the number of participants per Fitzpatrick skin type group: most (>85%) participants had skin types II–IV. In the other 6 studies, 5 specified that participants were mostly skin types III–IV and 1 study stated that participants were mostly skin types II–III.

Most image datasets (88%, *n* = 56) comprised macroscopic images of skin, hair or nails. Dermoscopic images were most commonly used for psoriasis (*n* = 5) and eczema (*n* = 4) (Table 1). In contrast to participant characteristics, the number of images used in training, validation and testing datasets was reported by most studies: 60 (93%), 61 (91%) and 62 (96%) studies, respectively. Generally, a greater number of images was used to train DL algorithms (median 2555 images, IQR 902–8550) than to validate (median 1032 images, IQR 274–2000) or test (median 331 images, IQR 157–922) DL algorithms. The ratio of median number of images to participants was 1.3 for training datasets, 1.6 for validation and 1.8 for testing datasets. This indicates that a single participant contributed more than one image through, for example, multiple photographs of anatomically distinct sites or splitting/modification of an image (Table 1).

### DL algorithms

**Fig. 3:**
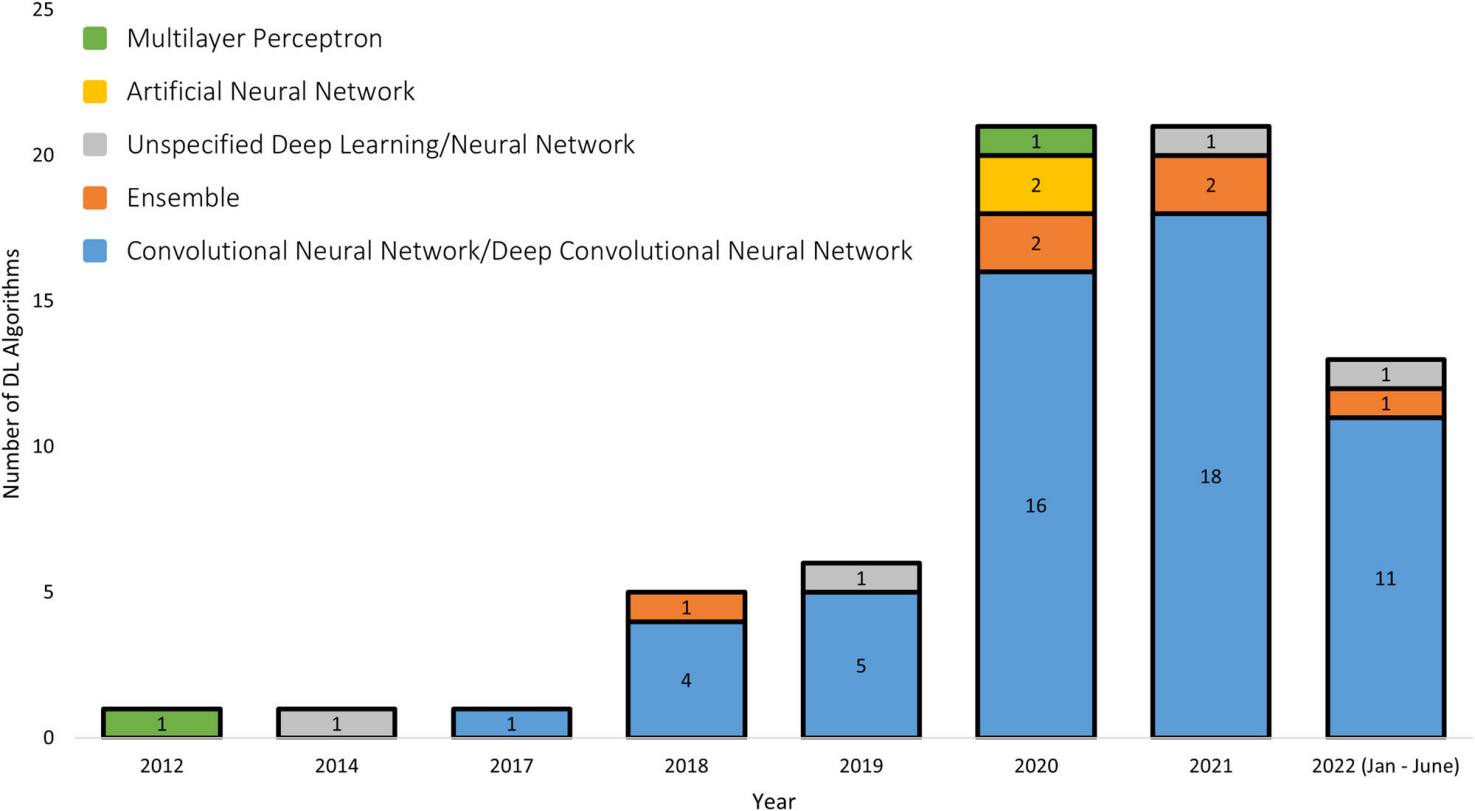
Type of deep learning algorithms included in the systematic review, by year of publication. The number of different types of deep learning (DL) algorithms are presented by year of publication of the studies. As five studies used multiple DL algorithms, the total number of algorithms sum to 69 across the 64 included studies.

Five studies used more than one type of DL algorithm, hence the total number of algorithms was 69 across 64 studies. Overall, the commonest types of DL algorithm were convolutional neural networks (CNN) and deep convolutional neural networks (DCNN) (80%, *n* = 55 of 69 algorithms) (Fig. 3 and Table 1). CNNs and DCNNs are considered interchangeable terms, as ‘deep’ refers to the number of layers in the algorithm architecture and most modern CNNs consist of a large number of layers^11^. The first CNN/DCNN study included in our review appeared in 2017. By 2021, 85% (*n* = 17 of 20) of studies applied CNN/DCNN algorithms. Ensemble DL algorithms, which combines multiple DL algorithms to improve prediction performance, first appeared in 2018, however was less frequently used compared to CNN/DCNN in subsequent years. Multilayer perceptron (MLP) (3%, *n* = 2 of 69 algorithms) and artificial neural networks (ANN) (3%, *n* = 2 of 69 algorithms), which are now considered outdated types of DL, were also less commonly used.

Most studies (77%, *n* = 49) reported the reference standard of the DL algorithm; 36 (73%) used a clinician assessment of images, of which 27 (75%) were dermatologists. The remaining 27% (*n* = 13 of 49) used multiple reference standards inconsistently across datasets or other reference standards including biopsies, blood tests and curated databases (Table 1). The severity scales used for disease severity grading DL algorithms were varied (Supplementary Material 8).

### Transparency

Most studies (95%, *n* = 61) disclosed the source of images. The most common sources of images were hospital/university databases (47%, *n* = 30), and many studies also used public databases (22%, *n* = 14). Image datasets were fully or partially available in under one third of studies (31%, *n* = 20). DL algorithm codes were available in 26 (41%) studies. In seven studies (11%) there were no details provided on the architecture of the DL algorithms (Table 1). With regards to transparency of reporting of primary and secondary outcomes, 26 of 64 studies (41%) provided the raw values that were used to calculate accuracy, sensitivity or specificity.

### Accuracy of diagnostic DL algorithms: six most studied diseases

Accuracy (the primary outcome) was the most commonly reported outcome for assessing the performance of all DL algorithms (75%, *n* = 48). The median diagnostic accuracy of the DL algorithms for the six most studied diseases (acne, psoriasis, eczema, rosacea, vitiligo, urticaria) was high, ranging from 81% for urticaria (*n* = 2) to 94% for both acne (IQR 86–98, *n* = 11) and rosacea (IQR 90–97, *n* = 4) (Table 2). The accuracies of the externally validated/tested diagnostic DL algorithms were higher for acne (median 92%, *n* = 2) and eczema (96%, *n* = 2) compared with psoriasis (74%, *n* = 1), however direct comparison was limited by the small number of studies. Most diagnostic DL algorithms for the six most studied diseases performed multiclass classification (79%, *n* = 26 of 33), rather than binary classification (21%, *n* = 7 of 33) (Supplementary Material 9).

### Accuracy of diagnostic DL algorithms: five categories of disease

The median diagnostic accuracy of DL algorithms for the five categories of skin diseases (inflammatory disorders, follicular disorders of skin, alopecia, pigmentary disorders, skin infections) was high, ranging from 88% for both skin infections (IQR 60–95, *n* = 17) and pigmentary disorders (IQR 80–99, *n* = 5) to 100% for alopecia (*n* = 2) (Table 3). The diagnostic accuracies of DL algorithms for inflammatory disorders (median 92%, IQR 80–96; *n* = 30) and follicular disorders of skin (median 93%, IQR 87–97; *n* = 16) were similarly high.

The median diagnostic accuracy of externally validated/tested DL algorithms was high for inflammatory disorders (83%, IQR 53–100; *n* = 6) and follicular disorders of skin (84%, *n* = 3), although numerically lower than that of all DL algorithms. Both studies reporting diagnostic accuracy of DL algorithms for alopecia used external testing and had an accuracy of 100%. In contrast, the median accuracy of externally validated/tested DL algorithms for diagnosing skin infections was low (59%, IQR 50–74; *n* = 7).

### Accuracy of severity grading DL algorithms

The analysis of DL algorithms for disease severity grading was limited by a paucity of studies (*n* = 12, Supplementary Material 8). The accuracy of DL algorithms in grading psoriasis severity was 93–100% (*n* = 2), however external validation/testing was not performed (Supplementary Material 10). The single study of a DL algorithm for grading eczema severity did perform external validation and reported 88% accuracy. Of 4 studies assessing DL algorithms that grade acne severity (median accuracy 76%, IQR 68-85), one performed external testing and reported lower accuracy (68%).

### Secondary outcomes of diagnostic DL algorithms: six most studied diseases

A total of 23 studies reported AUC. The median AUC of diagnostic DL algorithms was high, ranging from 0.90 (IQR 0.87–0.94, *n* = 4) for rosacea to 0.98 (IQR 0.93–0.99, *n* = 4) for acne (Table 2). Diagnostic accuracy of externally validated/tested DL algorithms was similarly high.

Overall, 29 studies reported specificity. The median specificity of diagnostic DL algorithms was high, ranging from 88% (IQR 80-98, *n* = 4) for vitiligo to 100% (*n* = 2) for urticaria (Table 2). Externally validated/tested algorithms had similarly high specificity, all above 96% (*n* = 6).

A total of 43 studies reported sensitivity. The median sensitivity of diagnostic DL algorithms was variable, ranging from 63% (IQR 42–92, *n* = 6) in rosacea to 91% (IQR 80-95, *n* = 5) in vitiligo. The range of sensitivity values for each disease was wide, and contrasted the narrower ranges for specificity. Externally validated/tested diagnostic DL algorithms generally had lower sensitivities compared with the overall dataset, ranging from 42% (*n* = 1) in rosacea to 87% (*n* = 2) in acne.

31 and 8 studies reported PPV and NPV, respectively. The median PPV of diagnostic DL algorithms varied from 77% for urticaria (*n* = 2) to 91% for vitiligo (*n* = 3) (Table 2). In contrast, the NPV of diagnostic DL algorithms was >90% for all six diseases, which was also a consistent finding for externally validated/tested DL algorithms.

### Secondary outcomes of diagnostic DL algorithms: five categories of disease

In line with the above findings, diagnostic DL algorithms for the five disease categories (inflammatory disorders, follicular disorders of skin, alopecia, pigmentary disorders, skin infections) were broadly highly specific but had variable sensitivity.

The median specificity of diagnostic DL algorithms ranged from 97% (IQR 93-99, *n* = 15) for follicular disorders of skin to 100% (*n* = 2) for alopecia (Table 3). With respect to inflammatory skin diseases (the most frequently studied disease category), the median accuracy of diagnostic DL algorithms was 98% (IQR 95-99, *n* = 40) and this remained high when only externally validated/tested algorithms were considered (100%, IQR 98-100; *n* = 10).

The median sensitivity of diagnostic DL algorithms ranged from 77% in inflammatory skin diseases (IQR 63–92, *n* = 47) and skin infections (IQR 63–93, *n* = 33) to 87% (IQR 67–94, *n* = 19) in follicular disorders (Table 3). When considering only externally validated/tested diagnostic DL algorithms, the median sensitivities remained variable and were lowest in inflammatory disorders (58%, IQR 48–72; *n* = 12) and skin infections (70%, IQR 56–80; *n* = 17), compared to follicular disorders (87%, IQR 63–92; *n* = 4). The range of sensitivity values for each disease category was also wide, and contrasted the narrower ranges for specificity.

### Secondary outcomes of severity grading DL algorithms

Although data were limited, the specificity of disease severity DL algorithms was high and ranged from 94–95% for acne (*n* = 2) to 97–100% for psoriasis (*n* = 2) (Supplementary Material 10). AUC was reported in only one study of a severity grading DL algorithm, in psoriasis (AUC 0.99). The sensitivity of disease severity grading DL algorithms ranged from 82–84% for acne (*n* = 2) to 93–96% for psoriasis (*n* = 2). PPV was reported for severity grading DL algorithms in acne (range 54–86%, *n* = 3) and psoriasis (93%, *n* = 1). No studies reported these metrics for externally validated/tested DL algorithms of disease severity (Supplementary Material 10).

## Discussion

This systematic review provides a comprehensive evaluation of DL image analysis studies of skin diseases. Skin conditions are the fourth leading cause of non-fatal disease burden worldwide^12^. Our review encompasses the commonest long-term skin conditions in the global population including eczema, psoriasis, acne, rosacea, vitiligo and urticaria. The reported diagnostic accuracy of DL algorithms was broadly encouraging for common inflammatory skin diseases, in contrast to skin infections, for which the diagnostic accuracy of externally validated/tested DL algorithms was generally low. Although diagnostic DL algorithms were mostly specific, there was variation in their sensitivities, which were notably lower in the fewer yet more robust studies that performed external validation or testing. While relatively few studies assessed DL algorithms for disease severity grading, the highest accuracy was reported in psoriasis, followed by eczema and acne.

Importantly, our findings on the reliability and applicability of current DL studies indicate that they should be interpreted with caution. There are key limitations, which bring the real-world clinical applicability of the reported DL algorithms into question. These include heterogeneity of study design and a lack of RCTs. Although 47% of studies utilised images from hospital or university databases, public databases were used in 22%, which may not be representative of the target population or healthcare setting of the algorithms’ intended use. The generalisability of the DL algorithms was also limited by poor capture of number, age, gender and skin colour of study participants. Ethnicity and/or Fitzpatrick skin type was reported in only 19% of studies and, amongst those reporting these characteristics, skin types I, V and VI were underrepresented. The reference standard for evaluating DL algorithm performance was inconsistent, with some studies not defining the ‘ground truth’ or specifying the type of clinician who assessed disease diagnosis/severity. There was also poor transparency of reporting of outcome metrics including confidence intervals for specificity and sensitivity, and numerator/denominator data. There were omissions in the reporting of data class-balance, and bias towards images of particular phenotypes and from specific geographical locations (Asia), leading to potentially skewed training datasets. Although authors implemented measures to mitigate model overfitting, the extent to which this was successful requires external validation/testing, which was only performed in 14% of studies.

There is a notable paucity of evidence synthesis of DL image analyses in non-cancer skin diseases, however similar limitations are highlighted in prior reviews of DL in skin cancer detection^8,13,14^, and are mirrored across other medical specialities at the forefront of DL image analysis research such as radiology and ophthalmology^15–19^. The need to improve the quality and interpretability of studies through improved reporting, consistent use of out-of-sample external validation/testing and well-defined clinical cohorts are common themes. A systematic review of 14 skin cancer image datasets similarly demonstrated poor ethnicity and/or Fitzpatrick skin type reporting^20^, which is in line with recent scoping reviews^21,22^. Most studies in our review were at early developmental stages with 84% stating that further prospective work or trials are required before clinical use. Notably, there are no current FDA approved AI/ML-enabled medical devices relating to dermatology, and of 521 total devices listed in the latest FDA update, most are applied to radiology (75%), followed by cardiovascular (11%) specialties^23^.

The strengths of this systematic review include a broad search strategy, which provides a comprehensive overview of DL image analysis in dermatology from its inception in the field. Our protocol, which adhered to PRISMA and SWiM guidelines, was developed with multidisciplinary input from experts in clinical dermatology, deep learning, and systematic reviews. Article searches, screening, data extraction and quality assessment were carried out by at least two independent researchers. We modified the QUADAS-2 tool to systematically assess the quality of included DL articles, which may be a valuable resource for future research in this rapidly expanding field.

Limitations include the lack of an existing formal quality assessment tool for AI/DL studies. Recent progress has been made, with PROBAST-AI^24^ and QUADAS-AI^25^ currently under development, in addition to publication of the CLEAR Derm consensus guidelines of best practice for image-based DL assessment^26^. Our modified QUADAS-2 tool is in line with the CLEAR Derm guidelines and enables in depth AI-centred evaluation of both risk of bias and applicability. We were unable to perform a meta-analysis due to the high degree of heterogeneity in the reporting of study design and outcome metrics. The small number of studies on each individual disease also precluded accurate inter-disease comparisons, in addition to variation in disease severity grading scales. Therefore, comparisons across studies should be interpreted with caution. Comparison of outcomes across diagnostic studies is particularly challenging since some DL algorithms perform binary classification and others perform multiclass classification. However, most diagnostic DL algorithms for the six most studied diseases in the systematic review performed multiclass classification, with few performing binary classification. Our search strategy was restricted to papers published in English, which may have omitted some studies, particularly given the dominance of investigators affiliated to China. To reduce result heterogeneity, we excluded studies that reported only results derived from pooling together data from non-lesional and lesional diagnoses; seven studies were excluded for this reason, suggesting that bias due to selective reporting^27^ may be an issue to be aware of in this evidence base. However, these exclusions may also have biased towards inclusion of studies of more simplistic DL algorithms.

Our findings are timely and clinically relevant. The recent prioritisation of teledermatology within healthcare workflows, enabling ready availability of skin images, has accelerated the need to understand the potential of DL image analysis. The deployment of AI technologies forms an integral part of national and international strategy to address the inequity of access to care for those with inflammatory skin conditions^28^. It may improve patient selection for early intervention by identifying those at risk of worse outcomes (severe disease), to help address the rising global burden of skin conditions. Our review indicates that DL image analysis has exciting potential, particularly in the diagnosis and severity assessment of common, highly treatable skin conditions (e.g. acne, eczema, psoriasis), however current studies have methodological and reporting concerns. There is a need for prospective image dataset curation with detailed clinical metadata, and external validation and testing of DL algorithms. Collaborative large-scale efforts from global dermatology networks^29,30^ to collect high-quality images from well-phenotyped cohorts are vital. A relative paucity of studies of disease severity versus diagnostic DL algorithms was also identified. Standardised reporting frameworks and performance evaluation metrics are critical for the interpretation of the wealth of emerging data. SPIRIT-AI and CONSORT-AI^31^ offer guidance for DL clinical trials. DL-specific reporting guidelines such as TRIPOD-AI^24,26,31^ and formalised regulatory, evaluation and data governance pathways^32^ will facilitate the transition of DL from the current experimental phase to realising its full potential in advancing healthcare efficiency and disease outcomes.

## Methods

### Search strategy and selection criteria

This systematic review is reported in accordance with the Preferred Reporting Items for Systematic Reviews and Meta-Analysis (PRISMA) guidelines^33^. The study protocol was prospectively registered on PROSPERO (CRD42022309935). The eligibility criteria were structured using the population, intervention, comparison, outcome, and study type (PICOS) framework (Supplementary Material 11)^34^. For population, we included skin, hair or nail diseases of any severity in all ages, ethnicities and skin types. Studies assessing wounds, and benign or malignant skin lesions were excluded. The intervention was DL algorithms applied to macroscopic and/or dermoscopic images of skin, hair or nail diseases to diagnose or grade the severity of disease. Although we included studies using any comparators, we defined our reference standard for evaluating DL algorithm performance as an assessment by a clinician. Studies using any outcome measures to report DL algorithm performance (e.g. accuracy, sensitivity, specificity) were included. Studies other than original research articles (e.g. letters, editorials, conference proceedings and reviews) were excluded.

We searched five electronic databases (PubMed, Embase, Web of Science, ACM digital library and IEEE Xplore). Search terms were selected based on consensus expert opinion and adapted for each database (Supplementary Material 12). All primary research articles published in peer-reviewed journals from 1^st^ January 2000 to 23^rd^ June 2022 were considered for inclusion.

Each study was screened using a two-stage process, using the systematic review web-tool Rayyan^35^. After removal of duplicates, five members of the research team (SPC, BJK, AP, WRT, SPT) independently screened titles and abstracts for potentially eligible studies so that each record was blindly assessed by at least two reviewers. Full texts of studies included from the initial screening stage were assessed for final eligibility by at least two researchers independently. Any disagreements were resolved by consensus or a third reviewer.

### Data analysis

Data from each included study were extracted by one member of the research team (SPC, BJK, AP, WRT, SMLL, JS, SPT) and checked by another member. Baseline characteristics, study design, characteristics and outcomes of DL algorithms were extracted using a predefined data extraction sheet. Any disagreements were resolved by consensus or a third reviewer. One author (BJK) amalgamated the extracted data and performed the data analysis.

Studies were categorised into diagnostic and severity grading studies. Diagnostic studies use DL to make predictions about disease diagnosis based on images. Severity studies make predictions about disease severity based on images, as measured by relevant severity scales e.g. psoriasis area and severity index (PASI) for psoriasis. Results were summarised separately for diagnosis and severity grading studies. Study types were categorised according to Prediction model Risk Of Bias Assessment Tool (PROBAST) definitions^36^ that we modified based on consensus among the research team to suit DL studies (Supplementary Material 13).

Given the large number of skin diseases covered in the review, we first summarised findings for the six most commonly studied diseases, followed by findings for five key skin disease categories (inflammatory disorders, follicular disorders of skin, alopecia, pigmentary disorders and skin infections). We referred to acne, rosacea and hidradenitis suppurativa as ‘follicular disorders of skin’ to distinguish them from alopecia, since studies of the latter use images of hair rather than skin.

To explore the impact of possible bias introduced by studies that use the same dataset to both train and validate/test algorithms, we also separately present the results for studies that use independent external data for validation and/or testing.

Due to high heterogeneity of the studies with respect to DL techniques and methods of evaluation, a meta-analysis would not have been suitable or informative. Instead, we conducted a narrative synthesis, following Synthesis Without Meta-analysis (SWiM) guidelines^37^. We performed descriptive statistical analyses, including calculation of median, interquartile range (IQR) and range using Microsoft Excel Version 2208. The primary outcome was the accuracy of DL algorithms in diagnosing and/or grading the severity of disease. Secondary outcomes included sensitivity, specificity, area under the receiver operating characteristic curve (AUC), positive predictive value (PPV) and negative predictive value (NPV).

### Quality assessment

There is a lack of quality assessment frameworks specific for AI/DL studies, with tools such as PROBAST-AI and QUADAS-AI still in development^24,25^. We modified the QUADAS-2 framework^25^ so that it could be used to assess risk of bias and applicability of DL studies in this systematic review and also provide a valuable resource for future research in the field (Supplementary Material 2). Questions were added to probe the robustness of DL algorithms in dermatology, such as the reporting of Fitzpatrick skin type, use of our defined reference standard (clinician assessment), and external validation/testing of the algorithm. All studies were blindly assessed using the modified QUADAS-2 by two reviewers (SPC, WRT) and any disagreements resolved through consensus.

### Reporting summary

Further information on research design is available in the Nature Research Reporting Summary linked to this article.

### Supplementary information

Reporting Summary

Supplementary Material

## Data Availability

All data supporting the findings of this study are available within the paper and its Supplementary Information files.
