# Supplementary material for: Systematic review of deep learning image analyses for the diagnosis and monitoring of skin disease
Source: NPJ Digit Med. 2023 Sep 27;6:180. doi: 10.1038/s41746-023-00914-8 (PMC10533565; doi:10.1038/s41746-023-00914-8)
Supplement: Supplementary file 2 — Supplementary Material [file 41746_2023_914_MOESM2_ESM.pdf]

Supplementary Material 1: Systematic review eligibility criteria

Study Component	Inclusion	Exclusion
Population	• All non-lesion dermatological disease (including skin, hair, and nail).<br>• All levels of disease severity<br>• Any age<br>• Any ethnic group<br>• Any skin type<br>• Application of the algorithm to both lesion and non-lesion skin diseases, when the results are clearly delineated such that the algorithm performance in non-lesion skin diseases is presented	• Animal studies<br>• Skin cancers, benign skin lesions<br>• Wounds, including diabetic/pressure ulcers and burns<br>• Conditions not relating to a particular skin disease (e.g. post-inflammatory hyper/hypo-pigmentation, photo-damaged skin, skin ageing, itchy skin, assessing image quality).<br>• Cancer treatment (e.g. chemotherapy induced alopecia, radiation induced dermatitis)<br>• Application of the algorithm to both lesion and non-lesion skin diseases, and reporting combined results without specifying performance in non-lesion skin diseases separately
Intervention	• Deep learning algorithms applied to macroscopic or dermoscopic skin images.	• Deep learning algorithms not applied to macroscopic or dermoscopic skin images (e.g. skin biopsy images, histology images, optical coherence tomography, diffuse spectroscopy, thermography, optoacoustic imaging, smartphone microscope images, fluorescence images, multispectral images)<br>• Machine learning is not a part of the intervention (e.g. image or signal enhancements, calibration and analysis only papers)<br>• Segmentation of images only, without diagnosis or severity assessment outcomes
Comparator	• Any, including clinician assessment, histopathological assessment, other machine learning algorithm performance.	
Outcome	• Best reported machine learning algorithm outcome, measured by any metrics, including accuracy, area under ROC, sensitivity, specificity, positive predictive value (PPV) and negative predictive value (NPV).	
Publication	• Published in English<br>• Year of publication (1st January 2000 to 23rd June 2022)<br>• Published in peer-reviewed journal	• Not original research<br>• Papers only published as abstracts<br>• No access to full article<br>• Conference proceedings
Study Design	• Comparative and non-comparative studies	• Non-original research articles (e.g. letters, conference posters, news articles, case report, case series)

Supplementary Material 2: Search strategies for bibliographic databases

PubMed Search Strategy

Search number	Keywords
1	skin disease*[MeSH Terms] OR skin disease*[Title/Abstract] OR dermatology[MeSH Terms] OR dermatology[Title/Abstract]
2	psoriasis[MeSH Terms] OR psoria*[Title/Abstract] OR pustulo*[Title/Abstract]) OR (palmopl*[Title/Abstract] OR palmari*[Title/Abstract] OR palmar[Title/Abstract]
3	eczema[MeSH Terms] OR eczema[Title/Abstract] OR atopic eczema[Title/Abstract] OR dermatitis[MeSH Terms] OR atopic dermatitis[Title/Abstract]
4	acne vulgaris[MeSH Terms] OR acne*[Title/Abstract] OR blackhead*[Title/Abstract] OR whitehead*[Title/Abstract] OR comedome*[Title/Abstract]
5	hidradenitis suppurativa[MeSH Terms] OR hidradenitis suppurativa[Title/Abstract] OR hydradenitis suppurativa[Title/Abstract] OR verneuil’s disease[Title/Abstract] OR velpeau’s disease[Title/Abstract] OR acne inversa[MeSH Terms] OR acne inversa[Title/Abstract] OR pyoderma fistulans significa[Title/Abstract] OR ectopic acne[Title/Abstract]
6	vitiligo [MeSH Terms] OR vitiligo[Title/Abstract] OR leucoderma[Title/Abstract] OR leukoderma[Title/Abstract] OR hypopigmentation[MeSH Terms] OR hypopigmentation[Title/Abstract] OR depigmentation[Title/Abstract]
7	1 OR 2 OR 3 OR 4 OR 5 OR 6
8	Title/Abstract: (Machine learning OR machine-learning OR artificial intelligence OR deep learning OR deep-learning OR convolutional neural network OR convolutional neural networks OR CNN OR smartphone app* OR computer vision OR neural network OR neural networks OR supervised learning OR unsupervised learning OR semi-supervised learning OR support vector machine OR support vector machines OR image segmentation OR semantic segmentation OR U-Net OR UNET OR k-means clustering OR k-nearest neighbors OR k-nearest neighbor) OR machine learning[MeSH] OR artificial intelligence[MeSH] OR deep learning[MeSH]
9	7 AND 8
10	Limit 9 to publications after 2000
11	Limit 10 to English-language publications
12	Deduplicate 11

Embase (Ovid SP) Search Strategy

Search number	Keywords
1	exp skin disease/ OR skin disease*.ab,ti. OR exp dermatology/ OR dermatology.ab,ti.
2	exp psoriasis/ OR psoria*.ab,ti. OR pustulo*.ab,ti. OR palmopl*.ab,ti. OR palmari*.ab,ti. OR palmar.ab,ti.
3	exp eczema/ OR eczema.ab,ti. OR atopic eczema.ab,ti. OR exp dermatitis/ OR atopic dermatitis.ab,ti.
4	exp acne vulgaris/ OR acne*.ab,ti. OR blackhead*.ab,ti. OR whitehead*.ab,ti. OR comedome*.ab,ti.
5	exp hidradenitis/ OR hidradenitis suppurativa.ab,ti. OR hydradenitis suppurativa.ab,ti. OR velpeau's disease.ab,ti. OR verneuil's disease.ab,ti. OR acne inversa.ab,ti. OR pyoderma fistulans significa.ab,ti. OR ectopic acne.ab,ti.
6	exp vitiligo/ OR vitiligo.ab,ti. OR leucoderma.ab,ti. OR leukoderma.ab,ti. OR exp hypopigmentation/ OR hypopigmentation.ab,ti. OR depigmentation.ab,ti.
7	1 OR 2 OR 3 OR 4 OR 5 OR 6
8	(Machine learning OR machine-learning OR artificial intelligence OR deep learning OR deep-learning OR convolutional neural network OR convolutional neural networks OR CNN OR smartphone app* OR computer vision OR neural network OR neural networks OR supervised learning OR unsupervised learning OR semi-supervised learning OR support vector machine OR support vector machines OR image segmentation OR semantic segmentation OR U-Net OR UNET OR k-means clustering OR k-nearest neighbors OR k-nearest neighbor).ab,ti. OR exp machine learning/ OR exp artificial intelligence/ OR exp deep learning/
9	7 AND 8
10	Limit 9 to publications after 2000
11	Limit 10 to English-language publications
12	Deduplicate 11

Web of Science Search Strategy

Search number	Keywords
1	TS=(skin disease* OR dermatology)
2	TS=(psoriasis) OR TI=(psoria* OR pustulo* OR palmopl* OR palmari* OR palmar) OR AB=(psoria* OR pustulo* OR palmopl* OR palmari* OR palmar)
3	TS=(eczema OR dermatitis) OR TI=(atopic eczema OR atopic dermatitis) OR AB=(atopic eczema OR atopic dermatitis)
4	TS=(acne vulgaris) OR TI=(acne* OR blackhead* OR whitehead* OR comedome*) OR AB=(acne* OR blackhead* OR whitehead* OR comedome*)
5	TS=(hidradenitis suppurative OR acne inversa) OR TI=(hydradenitis suppurative OR velpeau's disease OR verneuil's disease OR pyoderma fistulans significa OR ectopic acne) OR AB=(hydradenitis suppurative OR velpeau's disease OR verneuil's disease OR pyoderma fistulans significa OR ectopic acne)
6	TS=(vitiligo OR hypopigmentation) OR TI=(leucoderma OR leukoderma OR depigmentation) OR AB=(leucoderma OR leukoderma OR depigmentation)
7	1 OR 2 OR 3 OR 4 OR 5 OR 6
8	TS=(machine learning OR artificial intelligence OR deep learning) OR TI=(machine-learning OR convolutional neural network OR convolutional neural networks OR CNN OR deep-learning OR smartphone app* OR computer vision OR neural network OR neural networks OR supervised learning OR unsupervised learning OR semi-supervised learning OR support vector machine OR support vector machines OR image segmentation OR semantic segmentation OR U-Net OR UNET OR k-means clustering OR k-nearest neighbors OR k-nearest neighbor) OR AB=(machine-learning OR convolutional neural network OR convolutional neural networks OR CNN OR deep-learning OR smartphone app* OR computer vision OR neural network OR neural networks OR supervised learning OR unsupervised learning OR semi-supervised learning OR support vector machine OR support vector machines OR image segmentation OR semantic segmentation OR U-Net OR UNET OR k-means clustering OR k-nearest neighbors OR k-nearest neighbor)
9	7 AND 8
10	Limit 9 to publications after 2000
11	Limit 10 to English-language publications
12	Deduplicate 11

IEEE Search Strategy

Search number	Keywords
1	"Mesh_Terms":"skin disease" "Mesh_Terms":"skin diseases" OR "Mesh_Terms":dermatology OR (Publication Title:"skin disease" OR "skin diseases" OR dermatology)
2	"Mesh_Terms":"psoriasis" OR (Publication Title:psoria* OR "palmoplantar pustolosis" OR "pustulosis palmaris et plantaris") OR (Abstract:psoria* OR "palmoplantar pustolosis" OR "pustulosis palmaris et plantaris")
3	"Mesh_Terms":dermatitis OR "Mesh_Terms":eczema OR (Publication Title:"atopic eczema" OR "atopic dermatitis" OR eczema) OR (Abstract:"atopic eczema" OR "atopic dermatitis" OR eczema)
4	"Mesh_Terms":"acne vulgaris" OR (Publication Title:acne* OR "acne vulgaris" OR blackhead OR blackheads OR whitehead OR whiteheads OR comedome OR comedomes) OR (Abstract:acne* OR "acne vulgaris" OR blackhead OR blackheads OR whitehead OR whiteheads OR comedome OR comedomes)
5	"Mesh_Terms":"hidradenitis suppurativa" OR "Mesh_Terms":"acne inversa" (Publication Title: "hidradenitis suppurativa" OR "hydradenitis suppurativa" OR "acne inversa" OR "velpeau's disease" OR "verneuil's disease" OR "pyoderma fistulans significa" OR "ectopic acne") OR (Abstract: "hidradenitis suppurativa" OR "hydradenitis suppurativa" OR "acne inversa" OR "velpeau's disease" OR "verneuil's disease" OR "pyoderma fistulans significa" OR "ectopic acne")
6	"Mesh_Terms":vitiligo OR "Mesh_Terms":hypopigmentation OR (Publication Title: vitiligo OR hypopigmentation OR leucoderma OR leukoderma OR depigmentation) OR (Abstract: vitiligo OR hypopigmentation OR leucoderma OR leukoderma OR depigmentation)
7	1 OR 2 OR 3 OR 4 OR 5 OR 6
8	("Mesh_Terms":"machine learning" OR "Mesh_Terms":"artificial intelligence" OR "Mesh_Terms":"deep learning") OR (Publication Title:"Machine learning" OR "machine-learning" OR "artificial intelligence" OR "deep learning" OR "deep-learning" OR "convolutional neural network" OR "convolutional neural networks" OR CNN OR "smartphone app*" OR "computer vision" OR "neural network" OR "neural networks" OR "supervised learning" OR "unsupervised learning" OR "semi-supervised learning" OR "support vector machine" OR "support vector machines" OR "image segmentation" OR "semantic segmentation" OR "U-Net" OR "UNET" OR "k-means clustering" OR "k-nearest neighbors" OR "k-nearest neighbor") OR (Abstract:"Machine learning" OR "machine-learning" OR "artificial intelligence" OR "deep learning" OR "deep-learning" OR "convolutional neural network" OR "convolutional neural networks" OR CNN OR "smartphone app*" OR "computer vision" OR "neural network" OR "neural networks" OR "supervised learning" OR "unsupervised learning" OR "semi-supervised learning" OR "support vector machine" OR "support vector machines" OR "image segmentation" OR "semantic segmentation" OR "U-Net" OR "UNET" OR "k-means clustering" OR "k-nearest neighbors" OR "k-nearest neighbor")
9	7 AND 8
10	Limit 9 to publications after 2000
11	Deduplicate 10

ACM Digital Library Search Strategy

Search number	Keywords
1	Keyword:("skin disease*" OR dermatology) OR Title:("skin disease*" OR dermatology) OR Abstract:("skin disease*" OR dermatology)
2	Keyword:(psoriasis) OR Title:("psoria* OR pustulo* OR palmopl* OR palmari* OR palmar) OR Abstract:("psoria* OR pustulo* OR palmopl* OR palmari* OR palmar)
3	Keyword:(dermatitis OR eczema) OR Title:("dermatitis OR "atopic eczema" OR "atopic dermatitis" OR eczema) OR Abstract:("dermatitis OR "atopic eczema" OR "atopic dermatitis" OR eczema)
4	Keyword:("acne vulgaris") OR Title:("acne* OR "acne vulgaris" OR blackhead* OR whitehead* OR comedome*) OR Abstract:("acne* OR "acne vulgaris" OR blackhead* OR whitehead* OR comedome*)
5	Keyword:("hidradenitis suppurativa" OR "acne inversa") OR Title:("hidradenitis suppurativa" OR "hydradenitis suppurativa" OR "acne inversa" OR "velpeau's disease" OR "verneuil's disease" OR "pyoderma fistulans significa" OR "ectopic acne") OR Abstract:("hidradenitis suppurativa" OR "hydradenitis suppurativa" OR "acne inversa" OR "velpeau's disease" OR "verneuil's disease" OR "pyoderma fistulans significa" OR "ectopic acne")
6	Keyword:("vitilito OR hypopigmentation) OR Title:("vitiligo OR hypopigmentation OR leucoderma OR leukoderma OR depigmentation) OR Abstract:("vitiligo OR hypopigmentation OR leucoderma OR leukoderma OR depigmentation)
7	1 OR 2 OR 3 OR 4 OR 5 OR 6
8	Keyword:("machine learning" OR "artificial intelligence" OR "deep learning" OR Title:("Machine learning" OR "machine-learning" OR "artificial intelligence" OR "deep learning" OR "deep-learning" OR "convolutional neural network" OR "convolutional neural networks" OR CNN OR "smartphone app*" OR "computer vision" OR "neural network" OR "neural networks" OR "supervised learning" OR "unsupervised learning" OR "semi-supervised learning" OR "support vector machine" OR "support vector machines" OR "image segmentation" OR "semantic segmentation" OR "U-Net" OR "UNET" OR "k-means clustering" OR "k-nearest neighbors" OR "k-nearest neighbor") OR Abstract:("Machine learning" OR "machine-learning" OR "artificial intelligence" OR "deep learning" OR "deep-learning" OR "convolutional neural network" OR "convolutional neural networks" OR CNN OR "smartphone app*" OR "computer vision" OR "neural network" OR "neural networks" OR "supervised learning" OR "unsupervised learning" OR "semi-supervised learning" OR "support vector machine" OR "support vector machines" OR "image segmentation" OR "semantic segmentation" OR "U-Net" OR "UNET" OR "k-means clustering" OR "k-nearest neighbors" OR "k-nearest neighbor")
9	7 AND 8
10	Limit 9 to publications after 2000
11	Deduplicate 10

Supplementary Material 3: Modified PROBAST definitions for type of study

**Type of machine learning study**	**Definition**
Training only	Machine learning algorithm training (development) without external validation **and** external testing. These studies may include **internal** validation and/or internal testing, whereby algorithm performance was **only** evaluated with data used in the training process, including bootstrapping and cross-validation techniques.
Training and external validation	Machine learning algorithm training (development) combined with **external validation**, whereby algorithm performance was evaluated with independent data not used in the training process, and further adjustments could be made to the algorithm.
Training and external testing	Machine learning algorithm training (development) combined with **external testing**, whereby an unbiased, final evaluation of algorithm performance was conducted using independent data not used in the training process
External Testing only	**External** testing of existing (previously developed) algorithm with independent data not used in the training process.

Supplementary Material 4: Quality assessment methods - modified QUADAS-2 definitions and questions

Definitions

**Population**	For both diagnosis and severity studies, the population refers to one of the following types of clinical images datasets:<br><br>1. Self-developed datasets (recruited from patients within the study)<br>2. Self-developed datasets from previous publications<br>3. Open-sourced AND curated datasets<br>4. Open-sourced datasets (Online including Google searches)
**Reference standard**	For both diagnostic and severity studies, this is defined as expert diagnosis by a clinician, based on examination findings only.
**Index test**	Deep learning algorithms applied to macroscopic and dermoscopic skin images.
**Outcomes**	Metrics employed to evaluate deep learning algorithms in comparison to defined ground truth

Quality assessment

DOMAIN 1: PARTICIPANTS - RISK OF BIAS	
1) Were appropriate **data sources** used to obtain the datasets?<br><br>One of the following:<br><br>1. Self-developed datasets from patients recruited within the study<br>2. Well-curated, open-sourced image datasets<br>3. Previous publications with well-curated image datasets<br>4. Images obtained from clinical settings<br><br><u>Justification</u>: This question prompts the reader to determine if the paper employed data sources with well-developed datasets for their images to prevent risk-of-bias or errors.	**Yes** – if data sources were appropriate<br>Tick which is applicable:<br>☐ Self-developed datasets from patients recruited within the study<br>☐ Well-curated, open-sourced image datasets<br>☐ Previous publications with well-curated image datasets<br>☐ Images obtained from clinical settings<br><br>**No** – if data sources were **not** appropriate<br><br>**Unclear** – if source of datasets were unclear/not specified
2) Were **inclusions and exclusions of participants/images** appropriately reported<br>e.g., participant eligibility criteria / image search strategy and removal and/or criteria were **not over-restrictive**?<br><br><u>Justification</u>: This question prompts the reader to determine whether any inclusion or exclusion criteria, or the recruitment/image search strategy, could have made the included study participants unrepresentative of the intended target population **of the primary study.**<br><br>**<u>FURTHER PROMPTS</u>** Did the study avoid inappropriate exclusions, e.g.,<br>1. 'Difficult to diagnose' dermatoses/ presentations not excluded<br>2. Dermatoses/ presentations not excluded on basis of disagreement between evaluators	**Yes** – if participant eligibility criteria / image search strategy and removal were reported and/or criteria were not over-restrictive<br><br>**No** – if participant eligibility criteria / image search strategy and removal were **not** reported and/or criteria were over-restrictive<br><br>**Unclear** – if there was inadequate information provided on participant eligibility criteria / image search strategy and removal and/or unclear restriction of criteria
3) Was a **consecutive or random sample of patients/images** enrolled?<br><br><u>Justification</u>: To determine if the participants were randomly recruited or was there bias during the participant selection process.<br><br>Further prompts: This question prompts how strictly the description of the participant/image sampling was described.	**Yes –** if consecutive or random sampling was reported<br><br>**No –** if other method of sampling was reported<br><br>**Unclear –** if participant sampling not described
**Could the selection of participants have introduced bias?**<br><br>1: If answers to questions 1), 2) **AND** 3) were 'Yes'<br>2: If answers to questions 1) or 2) or 3) were 'No'<br>3: If answers to questions 1) or 2) or 3) were 'Unclear'	1. Risk is low<br>2. Risk is high<br>3. Risk is unclear

DOMAIN 1: PARTICIPANTS - CONCERN ABOUT APPLICABILITY	
1) Are the **included participants and chosen study setting generalisable to the patient population who will use the algorithm in practice**? * e.g., Fitzpatrick skin type<br><br>Justification: This question probes the clinical applicability of the algorithm to the demographics that it will be deployed for.<br><br>*This question is specific to the authors' intended real world clinical setting.	**Yes** – if study participants appear to be representative of the patient population that the algorithm was developed for<br><br>**No** – if study participants do **not** appear to be representative of the patient population that the algorithm was developed for<br><br>**Unclear** – if there is insufficient data to determine if the study participants appear to be representative of the patient population that the algorithm was developed for
2) Were **participant/data characteristics reported within the study** e.g., skin type, ethnicity, age, sex?<br><br>Justification – This question probes if the paper reports characteristics in sufficient detail for readers to understand the cohort that their algorithm is applicable to.	**Yes –** if participant/data characteristics were reported within the study with both the following minimum requirements:<br>- Skin type/Ethnicity<br>- Age<br><br>**No –** if participant/data characteristics were **not** reported within the study<br><br>**Unclear –** if insufficient data to determine if participant/data characteristics were reported within the study
3) Was an adequate **spectrum of disease subtypes/severity*** used to train the algorithm?<br><br>*Skin conditions with well-established severity grading system<br><br>Justification - This question probes the reader to check if there are a range of subtypes and/or severity for the datasets, so that it does not misrepresent or overfit to one severity/subtype.	**Yes –** if an adequate range of diagnoses/severity were included and reported<br><br>**No –** if an adequate range of diagnoses/severity were **not** included/not reported<br><br>**Unclear –** if there is insufficient data to the determine if there is an adequate range of diagnoses/severity
4) Was the employed **dataset class-balanced and/or were justifications provided for class imbalance for both training and testing dataset** to provide confidence/applicability of the algorithm's outcomes?<br><br>Justification – This question prompts the reader to determine if the dataset was class-balanced/justification (in terms of numbers within each class) for class-imbalance to provide confidence and applicability of the algorithm's outcomes.	**Yes** – if both the training and test set were class balanced/were justified for their imbalance<br><br>**No** – if both the training and test set were **not** class balanced/not justified for their imbalance<br><br>**Unclear** – if it's unclear as to whether the training and test set were class balanced/unclear justification for imbalance
**Is there concern that participants/images utilised do not reflect the patient population likely to be seen in clinical practice?**<br><br>1: If answers to questions 1), 2), 3) **AND** 4) were 'Yes'<br>2: If answers to any 1 of questions 1) or 2) or 3) or 4) were 'No'<br>3: If answers to any 1 of questions 1) or 2) or 3) or 4) were 'Unclear'	1. Concern is low<br>2. Concern is high<br>3. Concern is unclear

DOMAIN 2: REFERENCE STANDARD - RISK OF BIAS	
1) Is the **reference standard likely to correctly classify the target condition/severity** of the condition within the study?	**Yes** – if all participants with a final diagnosis/severity grading were assessed and/or verified by at least 1 dermatologist/clinician within the study<br><br>**No** – if all participants with a final diagnosis/severity grading were **not** assessed and/or verified by at least 1 dermatologist/clinician within the study<br><br>**Unclear** – if all participants with a final diagnosis/severity grading were assessed by non-medical personnel
2) If No to Question 1), is the **reference dataset sourced** from the following sources:<br><br>1. Well-curated, open-sourced image datasets<br>2. Previous publications with well-curated image datasets<br>3. Images obtained from clinical settings but not verified by an independent dermatologist/clinician<br><br>*Note if there are multiple datasets, report the dataset with the **<u>highest risk</u>** only.	**Yes** - if dataset was sourced from 1 of the 3 sources defined<br><br>**No** – if dataset was **not** sourced from any of the 3 sources defined<br><br>**Unclear** – if unclear as to whether dataset was curated
**Could the reference standard, its conduct, or interpretation have introduced bias?**<br><br>1: If answer to either question 1) or 2) was 'Yes'<br>2: If answers to both questions 1) **AND** 2) were 'No'<br>3: If answers to either questions 1) OR 2) were 'Unclear'	1. Risk is low<br>2. Risk is high<br>3. Risk is unclear

DOMAIN 3: INDEX TEST – Risk of bias	
1) Were algorithm **overfitting, under-fitting, and optimism in algorithm performance** accounted for?<br><br>For example, do they mention use of any of the following:<br>a. Hold-out/Train-test split<br>b. Cross-validation<br>c. Data augmentation<br>d. L1/L2 Regularisation<br>e. Removal of layers<br>f. Dropout layers<br>g. Early stopping<br>h. Ensembling<br>i. Class balance	**Yes** – if algorithm overfitting, under-fitting, and optimism in algorithm performance was accounted for<br>Tick the following if applicable:<br>☐ Hold-out/Train-test split<br>☐ Cross-validation<br>☐ Data augmentation<br>☐ L1/L2 Regularisation<br>☐ Removal of layers<br>☐ Dropout layers<br>☐ Early stopping<br>☐ Ensembling<br>☐ Class balance<br><br>**No** – if algorithm overfitting, under-fitting, and optimism in algorithm performance were **not** accounted for<br><br>**Unclear** – if it is not clearly reported on algorithm performance
**Is there risk of introducing bias in the conduct and interpretation of the index test?**<br><br>1: If answers to question 1) was 'Yes'<br>2: If answers to question 1) was 'No'<br>3: If answers to question 1) was 'Unclear'	1. Risk is low<br>2. Risk is high<br>3. Risk is unclear

DOMAIN 3: INDEX TEST – CONCERN ABOUT APPLICABILITY	
1) Was the **deep learning algorithm and available dataset sufficient to allow for replication?**<br><br>E.g., if source code/datasets were available to replicate their results, and if **ALL** the outcomes were provided for others to replicate their results.<br><br><u>Justification</u> – This question prompts the reader to determine if the source code/datasets are available for them to replicate the same outcomes.	**Yes** – if the algorithm and presented dataset were reported in sufficient details to allow for replication<br><br>**No** – if the algorithm and presented dataset were **not** reported in sufficient details to allow for replication<br><br>**Unclear** – if it is unclear that the algorithm and presented dataset were reported in sufficient details to allow for replication
2) Has the **algorithm(s) been evaluated on an independent dataset (external validated/ tested), in a clinical setting (e.g., compared against dermatologists/ specialists), or in a prospective clinical trial with the intended population?**	**Yes** – if the algorithm(s) has been evaluated on an independent dataset (external validated/ tested), in a clinical setting (e.g., compared against dermatologists/ specialists), or in a prospective clinical trial with the intended population<br><br>**No** – if the algorithm(s) has **not** been evaluated on an independent dataset (external validated/ tested), in a clinical setting (e.g., compared against dermatologists/ specialists), or in a prospective clinical trial with the intended population<br><br>**Unclear** – if it is unclear if the algorithm(s) has been evaluated on an independent dataset (external validated/ tested), in a clinical setting (e.g., compared against dermatologists/ specialists), or in a prospective clinical trial with the intended population
**Do the index tests have concerns about applicability?**<br><br>1: If answers to both questions 1) **AND** 2) were 'Yes'<br>2: If answers to either questions 1) or 2) were 'No'<br>3: If answers to either questions 1) or 2) were 'Unclear'	1. Concern is low<br>2. Concern is high<br>3. Concern is unclear

DOMAIN 4: OUTCOMES AND ANALYSIS – RISK OF BIAS	
1) Were all collected images in the dataset included* in the analysis?<br><br>*E.g., Sampling of control participants, image manipulation (removal of hair from image, anonymising images (tattoos, faces, eyes), restricting to certain body regions/angle, removal of artefacts (text box, annotations))	**Yes** – if all collected images in the dataset were included in the analysis<br><br>**No** – if all collected images in the dataset were **not** included in the analysis<br><br>**Unclear** – if it is unclear if all collected images in the dataset were included in the analysis
2) Was the **appropriate metric(s) of the index test outcome(s) defined/determined and equally applied to all participants/algorithms**?<br><br>i.e. Standard metrics (e.g., accuracy, along with sensitivity and specificity) used **AND** reported.<br><br><u>Justification</u> – This question prompts the reader to determine if the metrics were appropriate and accurately reported (e.g., formula, no discrepancies across figures and text) and were applied to all participants/algorithms (i.e., authors described methods and reported in results).	**Yes** – if metric(s) of the outcome(s) was defined/determined appropriately and equally applied to all participants/algorithms<br><br>**No** – if metric(s) of the outcome(s) was **not** defined/determined appropriately and equally applied to all participants/algorithms<br><br>**Unclear** – if it is unclear about the metric(s) of the outcome(s) is defined/determined appropriately and equally applied to all participants/algorithms
3) Were relevant **measures of variability reported appropriately?** i.e. <u>reporting of metrics with CI/SD/SEM</u><br><br><u>Justification</u> – This question prompts the reader to evaluate if the CI/SD/SEM for all the metrics is reported, which provides an unbiased evaluation of the metric and of its validity.	**Yes** – if relevant measures of variability were reported appropriately<br><br>**No** – if relevant measures of variability were **not** reported appropriately<br><br>**Unclear** – if it is unclear if relevant measures of variability were reported appropriately
**Is there risk of introducing bias in the conduct and interpretation of the outcome and analysis?**<br><br>1: If answers to questions 1), 2) **AND** 3) were 'Yes'<br>2: If answers to any 1 of questions 1) or 2) or 3) were 'No'<br>3: If answers to any 1 of questions 1) or 2) or 3) were 'Unclear'	1. Risk is low<br>2. Risk is high<br>3. Risk is unclear

DOMAIN 4: OUTCOMES AND ANALYSIS – CONCERN ABOUT APPLICABILITY	
1) Was the **study outcome(s) clearly defined and determined appropriately?**<br><br><u>Justification</u> – This question prompts the reader to determine if the study outcome(s) were clearly defined and the determination of these outcomes were appropriate to answer the study's hypothesis.	**Yes** – if the study outcome(s) was clearly defined and determined appropriately<br><br>**No** – if the study outcome(s) was **not** clearly defined and determined appropriately<br><br>**Unclear** – if there was lack of clarity in the definition and determination of study outcome(s)
**Is there concern that the outcome and analysis have introduced bias?**<br><br>1: If answers to question 1) was 'Yes'<br>2: If answers to question 1) was 'No'<br>3: If answers to question 1) was 'Unclear'	1. Concern is low<br>2. Concern is high<br>3. Concern is unclear

Supplementary Material 5: Quality assessment results, by study (using modified QUADAS-2)

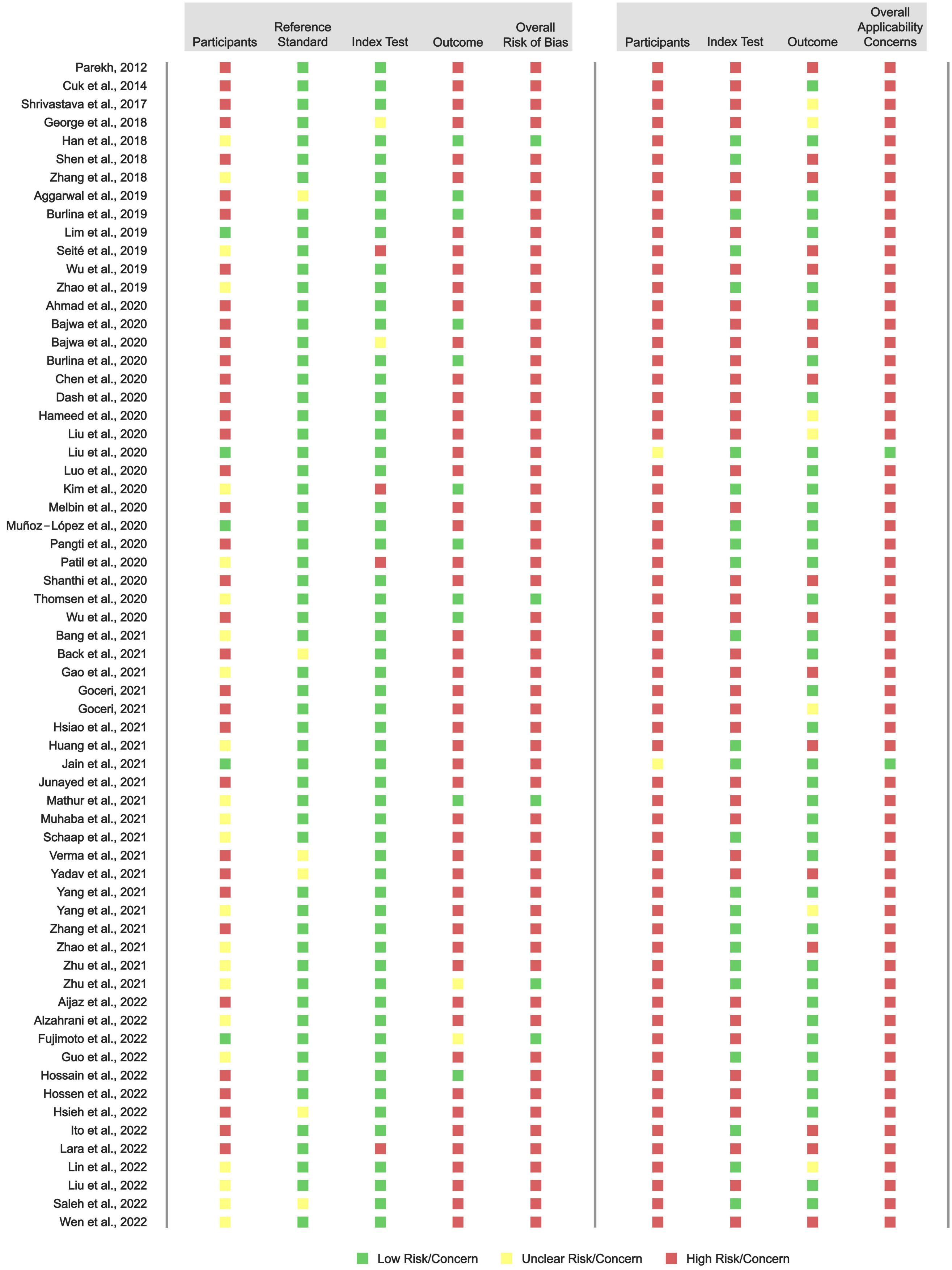

Studies are ordered chronologically, then alphabetically.

Supplementary Material 6: Summary of quality assessment results of studies of externally validated/tested deep learning algorithms (using modified QUADAS-2)

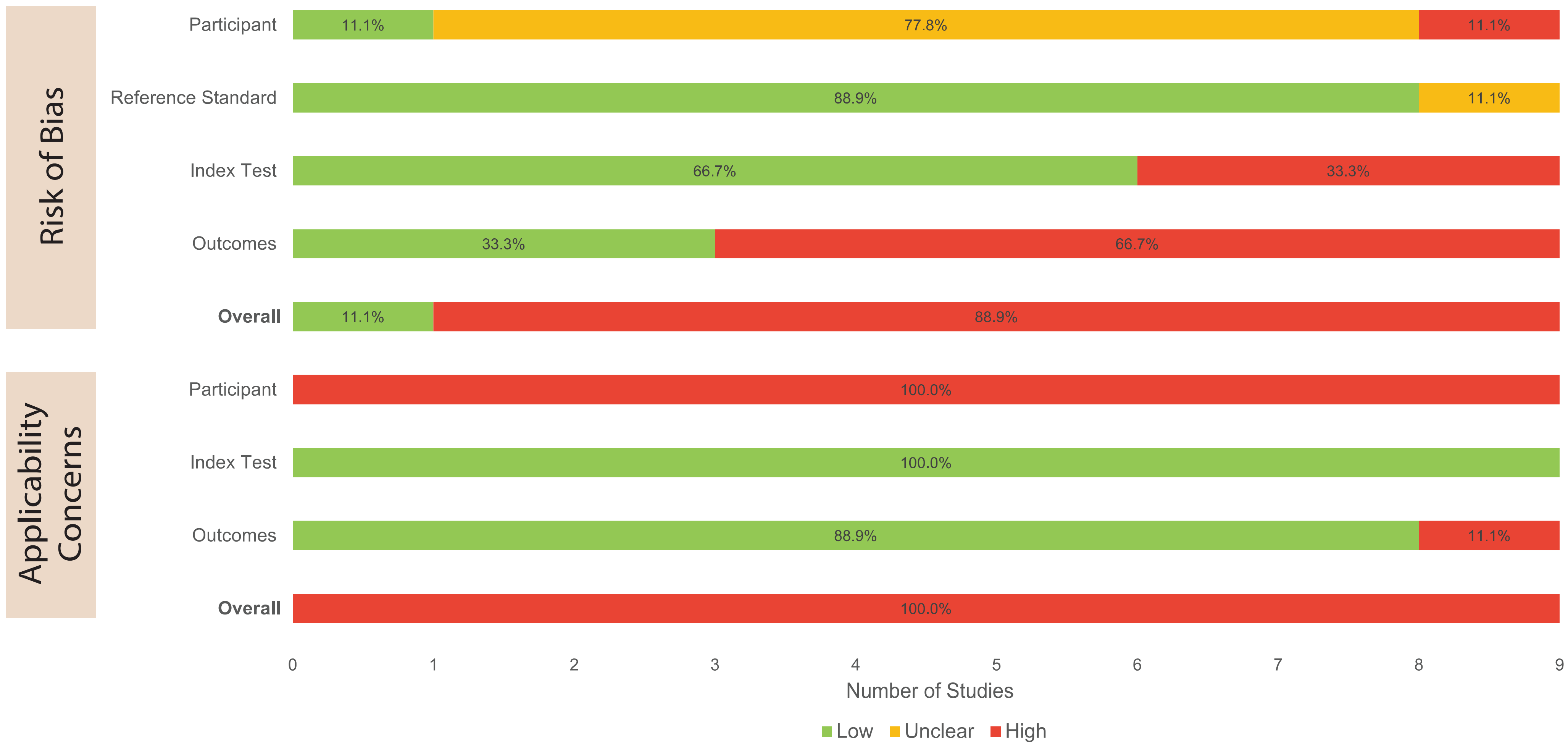

Supplementary Material 7: Funding for studies

Funding status	Number of studies (total n=64)
Received funding	47 (73.4%)
No funding	6 (9.4%)
Unclear	11 (17.2%)

Supplementary Material 8: Geographical region for affiliation of authors and source of private datasets

Country of affiliation	Number of studies
China	20
India	9
USA	5
Asia - other	13
Europe	7
Middle East	4
South America	2
Mixed	2
Australia	1
Africa	1
Total	64

Geographic source of private datasets	Number of studies
China	18
India	7
Asia - other	10
Europe	5
North America	4
Africa	2
South America	1
Australia	1
Total	48

Countries with five or more studies are listed individually at the top of the table in descending order of frequency. Countries with less than five studies are grouped into geographical regions in descending order of frequency. 'Asia - other' comprises Bangladesh, Japan, Singapore, South Korea and Taiwan.

Supplementary Material 9: Baseline characteristics and outcomes for studies of externally validated/tested deep learning algorithms

Author	Year	Disease	Type of study	Study design	Function of DL algorithm	Total no. of images**	Reference standard	Use of 2-by-2 matrix/ confusion matrix	Internal dataset: Sensitivity (95% CI or SD)	Internal dataset: Specificity (95% CI or SD)	External dataset: Sensitivity (95% CI or SD)	External dataset: Specificity (95% CI or SD)
**Studies of single disease**												
Bang et al.	2021	Eczema	Training and External Validation	Retrospective	Severity	7600	Dermatologist(s)	Yes	NR	NR	NR	NR
Han et al.	2018	Onychomycosis	Training and External Testing	Retrospective	Diagnosis	26993.5	Dermatologist(s)	No	96.0% (± 0.0)	98.0% (± 0.0)	96.0% (± 0.0)	94.7% (± 2.3)
Kim et al.	2020	Onychomycosis	External Testing only	Prospective	Diagnosis	NR	Mixed - Dermatologist(s) + Dermoscopy + KOH studies	No	NR	NR	70.2% (NR)	72.7% (NR)
Seité et al.	2019	Acne	Training and External Testing	Retrospective	Severity	5972	Dermatologist(s)	No	NR	NR	NR	NR
Guo et al.	2022	Vitiligo	Training and External Testing	Retrospective	Diagnosis	2030.5	Dermatologist(s)	No	92.9% (NR)	NR	72.4% (NR)	NR
**Studies of multiple diseases**												
Muñoz-López et al.	2020	Multiple*	External Testing only	Retrospective	Diagnosis	322.5	Dermatologist(s)	No	NR	NR	NR	NR
Pangti et al.	2020	Multiple*	Training and External Testing	Retrospective	Diagnosis	27768	Mixed - Curated Database(s) + Dermatologist(s)	Yes	NR	NR	See below	See below
		Acne							NR	NR	86.23% (± 3.26)	99.56% (± 0.13)
		Eczema							NR	NR	57.52% (± 3.37)	99.00% (± 0.12)
		Psoriasis							NR	NR	68.00% (± 5.06)	99.18% (± 0.13)
		Rosacea							NR	NR	90.17% (± 4.40)	99.62% (± 0.13)
		Urticaria							NR	NR	70.10% (± 7.58)	99.91% (± 0.04)
		Vitiligo/ Leucoderma							NR	NR	84.10% (± 6.84)	99.79% (± 0.11)
Patil et al.	2020	Multiple*	External Testing only	Prospective	Diagnosis	348	Dermatologist(s)	Yes	NR	NR	See below	NR
		Acne							NR	NR	84.0% (NR)	NR
		Eczema							NR	NR	91.7% (NR)	NR
		Psoriasis							NR	NR	73.7% (NR)	NR
Saleh et al.	2022	Multiple*	Training and External Testing	Retrospective	Diagnosis	40200	Unavailable	Yes	NR	NR	NR	NR

Of 64 included studies, 9 studies used external datasets to validate and/or test their DL algorithms (i.e. datasets independent from the training dataset). The baseline characteristics and outcomes of these "externally validated/tested studies", presumed to be at a lower risk of overfitting, are presented. Where studies report multiple results by using variations of DL algorithms or datasets, the best performing results are presented. Where studies use both internal and external datasets to validate and/or test their DL algorithms, outcomes are presented separately for comparison.

*For studies with multiple diseases, only the outcomes of the six most frequently studied diseases (acne, psoriasis, eczema, rosacea, vitiligo, urticaria) are presented in this table.
**Total number of images used across all datasets (training, validation, testing)
NR, not reported; CI, confidence interval; SD, standard deviation; KOH, potassium hydroxide.

Supplementary Material 10: Disease severity scales employed in studies of deep learning algorithms

Author	Year	Disease	Disease severity scale
Lim et al.	2019	Acne	IGA
Lin et al.	2022	Acne	Hayashi criterion / Pillsbury criterion
Liu et al.	2022	Acne	Hayashi criterion
Seité et al.	2019	Acne	European GEA Scale
Wen et al.	2022	Acne	Hayashi criterion
Yang et al.	2021	Acne	Chinese AGS
Gao et al.	2021	Androgenetic Alopecia	BASP classification
Bang et al.	2021	Eczema	EASI
Dash et al.	2020	Psoriasis	PGA
George et al.	2018	Psoriasis	Erythema severity score
Schaap et al.	2021	Psoriasis	PASI
Shrivastava et al	2017	Psoriasis	PGA
Total			12

Acne Grading System, AGS; Basic and Specific Classification of androgenic hair loss, BASP; Eczema Area and Severity Index, EASI; Global Acne Severity Scale, GEA Scale; Investigator Global Assessment, IGA; Psoriasis Area Severity Index, PASI; Physician Global Assessment, PGA.

Supplementary Material 11: Outcomes of binary and multiclass deep learning algorithms for the diagnosis of the six most studied diseases

	Outcome											
	**Accuracy (%)**		**AUC**		**Sensitivity (%)**		**Specificity (%)**		**PPV (%)**		**NPV (%)**	
Type of algorithm	Binary	Multiclass	Binary	Multiclass	Binary	Multiclass	Binary	Multiclass	Binary	Multiclass	Binary	Multiclass
**Acne**												
**Median (IQR)**	97.5 (n/a)	93.0 (85.7 - 95.2)	n/a	0.98 (0.93 - 0.99)	n/a	89.9 (82.2 - 96.3)	n/a	95.2 (92.9 - 97.6)	n/a	86.5 (81.3 - 87.5)	n/a	96.0 (n/a)
**Range**	97.5	79.0 - 99.7	n/a	0.89 - 0.99	n/a	67.0 - 100.0	n/a	92.1 - 100.0	n/a	78.6 - 100.0	n/a	93.4 - 98.6
**Number of studies**	1	10	0	4	0	11	0	8	0	10	0	2
**Psoriasis**												
**Median (IQR)**	n/a	89.1 (78.1 - 92.0)	0.98 (n/a)	0.90 (0.84 - 0.96)	92.5 (n/a)	83.2 (70.2 - 91.7)	96.6 (n/a)	93.3 (89.2 - 96.1)	n/a	82.4 (60.6 - 88.6)	n/a	94.8 (n/a)
**Range**	n/a	69.4 - 98.5	0.98	0.81 - 0.99	92.0 - 92.9	60.0 - 95.6	95.2 - 98.0	88.2 - 98.8	n/a	60 - 95.5	n/a	91.5 - 98.1
**Number of studies**	0	8	1	4	2	8	2	6	0	7	0	2
**Eczema**												
**Median (IQR)**	n/a	92.6 (89.7 - 99.4)	n/a	0.93 (0.87 - 0.99)	77.3 (n/a)	87.8 (70.2 - 94.6)	92.6 (n/a)	97.2 (91.0 - 99.1)	n/a	77.1 (61.9 - 89.7)	n/a	93.2 (n/a)
**Range**	n/a	83.9 - 99.9	n/a	0.79 - 0.99	77.3	54.3 - 99.6	92.6	86.6 - 99.6	n/a	43.0 - 98.9	n/a	90.5 - 95.8
**Number of studies**	0	9	0	6	1	12	1	9	0	8	0	2
**Rosacea**												
**Median (IQR)**	n/a	93.7 (89.6 - 96.9)	n/a	0.90 (0.87 - 0.94)	n/a	63.4 (41.7 - 92.0)	n/a	97.0 (93.9 - 99.3)	n/a	89.8 (35.7 - 94.5)	n/a	95.1 (n/a)
**Range**	n/a	87.8 - 97.9	n/a	0.85 - 0.97	n/a	0.0 - 100.0	n/a	91.7 - 99.8	n/a	0.0 - 95.0	n/a	90.2 - 99.9
**Number of studies**	0	4	0	4	0	6	0	5	0	7	0	2
**Vitiligo**												
**Median (IQR)**	86.8 (n/a)	100 (n/a)	0.97 (n/a)	0.98 (n/a)	89.7 (80.4 - 94.1)	92.9 (n/a)	80.2 (n/a)	98.8 (n/a)	91.4 (n/a)	80.1 (n/a)	n/a	99.6 (n/a)
**Range**	85.7 - 87.8	100	0.94 - 1.00	0.98	72.4 - 97.2	92.9	79.4 - 96.3	98.8	90.9 - 91.9	80.1	n/a	99.6
**Number of studies**	2	1	2	1	4	1	3	1	2	1	0	1
**Urticaria**												
**Median (IQR)**	n/a	80.6 (n/a)	n/a	0.91 (n/a)	n/a	65.8 (n/a)	n/a	99.8 (n/a)	n/a	76.9 (n/a)	n/a	99.5 (n/a)
**Range**	n/a	68.3 - 92.8	n/a	0.91	n/a	55.7 - 75.9	n/a	99.7 - 99.8	n/a	75.6 - 78.2	n/a	99.5
**Number of studies**	0	2	0	1	0	2	0	2	0	2	0	1

The six most studied diseases are acne, psoriasis, eczema, rosacea, vitiligo and urticaria. Studies assessing multiple diseases are reported in each of the relevant disease columns. Where studies report multiple outcomes by using variations of DL algorithms or datasets, the best performing results are presented. Interquartile ranges (IQR) are not presented for less than four studies.

Deep learning, DL; area under the receiver operating characteristic curve, AUC; positive predictive value, PPV; negative predictive value, NPV; interquartile range, IQR.

Supplementary Material 12: Outcomes of deep learning algorithms for the assessment of skin disease severity

	Outcome											
	Accuracy (%)		AUC		Sensitivity (%)		Specificity (%)		PPV (%)		NPV (%)	
	All studies	Externally validated/tested studies	All studies	Externally validated/tested studies	All studies	Externally validated/tested studies	All studies	Externally validated/tested studies	All studies	Externally validated/tested studies	All studies	Externally validated/tested studies
**Acne**												
**Median (IQR)**	76.3 (67.5 - 85.2)	68.0 (n/a)	n/a	n/a	82.9 (n/a)	n/a	94.4 (n/a)	n/a	83.6 (n/a)	n/a	n/a	n/a
**Range**	67 - 85.8	68.0	n/a	n/a	82.0 - 83.7	n/a	94.1 - 94.6	n/a	53.6 - 85.6	n/a	n/a	n/a
**Number of studies**	4	1	0	0	2	0	2	0	3	0	0	0
**Psoriasis**												
**Median (IQR)**	96.2 (n/a)	n/a	0.99 (n/a)	n/a	94.3 (n/a)	n/a	98.6 (n/a)	n/a	92.7 (n/a)	n/a	n/a	n/a
**Range**	92.6 - 99.7	n/a	0.99	n/a	92.6 - 95.9	n/a	97.4 - 99.7	n/a	92.7	n/a	n/a	n/a
**Number of studies**	2	0	1	0	2	0	2	0	1	0	0	0
**Eczema**												
**Median (IQR)**	88.3 (n/a)	88.3 (n/a)	n/a	n/a	n/a	n/a	n/a	n/a	n/a	n/a	n/a	n/a
**Range**	88.3	88.3	n/a	n/a	n/a	n/a	n/a	n/a	n/a	n/a	n/a	n/a
**Number of studies**	1	1	0	0	0	0	0	0	0	0	0	0

Studies of deep learning algorithms assessing multiple diseases were reported under each of the relevant diseases. Where studies report multiple outcomes by using variations of deep learning (DL) algorithms or datasets, the best performing results are presented. Outcomes for “externally validated/tested studies” (i.e. where datasets independent from the training dataset were used for validation and/or testing DL algorithms) are presented separately from “all studies”, as these studies are presumed to be at a lower risk of overfitting. Interquartile ranges (IQR) are not presented for less than four studies.

Deep learning, DL; area under the receiver operating characteristic curve, AUC; positive predictive value, PPV; negative predictive value, NPV; interquartile range, IQR.

Supplementary Material 13: Reference list of all included studies

1. Aggarwal, 1stLP. Data augmentation in dermatology image recognition using machine learning. *Skin Research and Technology* 2019; **25**(6): 815-20.
2. Ahmad B, Usama M, Huang C-M, Hwang K, Hossain MS, Muhammad G. Discriminative feature learning for skin disease classification using deep convolutional neural network. *IEEE Access* 2020; **8**: 39025-33.
3. Aijaz SF, Khan SJ, Azim F, Shakeel CS, Hassan U. Deep learning application for effective classification of different types of psoriasis. *Journal of Healthcare Engineering* 2022; **2022**.
4. Alzahrani S, Al-Bander B, Al-Nuaimy W. Attention mechanism guided deep regression model for acne severity grading. *Computers* 2022; **11**(3): 31.
5. Back S, Lee S, Shin S, et al. Robust skin disease classification by distilling deep neural network ensemble for the mobile diagnosis of herpes zoster. *IEEE Access* 2021; **9**: 20156-69.
6. Bajwa MN, Muta K, Malik MI, et al. Computer-aided diagnosis of skin diseases using deep neural networks. *Applied Sciences* 2020; **10**(7): 2488.
7. Bajwa UI, Alam S, Ratyal NI, Anwar MW. Skin disease classification using neural network. *Current Medical Imaging* 2020; **16**(6): 711-9.
8. Bang CH, Yoon JW, Ryu JY, et al. Automated severity scoring of atopic dermatitis patients by a deep neural network. *Scientific Reports* 2021; **11**(1): 6049.
9. Burlina PM, Joshi NJ, Mathew PA, Paul W, Rebman AW, Aucott JN. AI-based detection of erythema migrans and disambiguation against other skin lesions. *Computers in biology and medicine* 2020; **125**: 103977.
10. Burlina PM, Joshi NJ, Ng E, Billings SD, Rebman AW, Aucott JN. Automated detection of erythema migrans and other confounding skin lesions via deep learning. *Computers in biology and medicine* 2019; **105**: 151-6.
11. Chen M, Zhou P, Wu D, Hu L, Hassan MM, Alamri A. AI-Skin: Skin disease recognition based on self-learning and wide data collection through a closed-loop framework. *Information Fusion* 2020; **54**: 1-9.
12. Čuk E, Gams M, Možek M, Strle F, Čarman VM, Tasič JF. Supervised visual system for recognition of erythema migrans, an early skin manifestation of lyme borreliosis. *Strojniški vestnik-Journal of Mechanical Engineering* 2014; **60**(2): 115-23.
13. Dash M, Londhe ND, Ghosh S, Raj R, Sonawane RS. A cascaded deep convolution neural network based CADx system for psoriasis lesion segmentation and severity assessment. *Applied Soft Computing* 2020; **91**: 106240.
14. Fujimoto A, Iwai Y, Ishikawa T, et al. Deep neural network for early image diagnosis of Stevens-Johnson syndrome/toxic epidermal necrolysis. *The Journal of Allergy and Clinical Immunology: In Practice* 2022; **10**(1): 277-83.
15. Gao M, Wang Y, Xu H, et al. Deep Learning-based Trichoscopic Image Analysis and Quantitative Model for Predicting Basic and Specific Classification in Male Androgenetic Alopecia. *Acta Dermato-Venereologica* 2022; **102**: adv00635-adv.
16. George Y, Aldeen M, Garnavi R. Psoriasis image representation using patch-based dictionary learning for erythema severity scoring. *Computerized Medical Imaging and Graphics* 2018; **66**: 44-55.
17. Goceri E. Deep learning based classification of facial dermatological disorders. *Computers in Biology and Medicine* 2021; **128**: 104118.
18. Goceri E. Diagnosis of skin diseases in the era of deep learning and mobile technology. *Computers in Biology and Medicine* 2021; **134**: 104458.
19. Guo L, Yang Y, Ding H, et al. A deep learning-based hybrid artificial intelligence model for the detection and severity assessment of vitiligo lesions. *Annals of Translational Medicine* 2022; **10**(10).
20. Hameed N, Shabut AM, Ghosh MK, Hossain MA. Multi-class multi-level classification algorithm for skin lesions classification using machine learning techniques. *Expert Systems with Applications* 2020; **141**: 112961.
21. Han SS, Park GH, Lim W, et al. Deep neural networks show an equivalent and often superior performance to dermatologists in onychomycosis diagnosis: Automatic construction of onychomycosis datasets by region-based convolutional deep neural network. *PloS one* 2018; **13**(1): e0191493.
22. Hossain SI, de Herve JdG, Hassan MS, et al. Exploring convolutional neural networks with transfer learning for diagnosing Lyme disease from skin lesion images. *Computer Methods and Programs in Biomedicine* 2022; **215**: 106624.
23. Hossen MN, Panneerselvam V, Koundal D, Ahmed K, Bui FM, Ibrahim SM. Federated machine learning for detection of skin diseases and enhancement of internet of medical things (IoMT) security. *IEEE journal of biomedical and health informatics* 2022.
24. Hsiao Y-P, Chiu C-W, Lu C-W, et al. Identification of skin lesions by using single-step multiframe detector. *Journal of Clinical Medicine* 2021; **10**(1): 144.
25. Hsieh KY, Chen H-Y, Kim S-C, Tsai Y-J, Chiu H-Y, Chen G-Y. A mask R-CNN based automatic assessment system for nail psoriasis severity. *Computers in Biology and Medicine* 2022; **143**: 105300.
26. Huang K, Jiang Z, Li Y, et al. The Classification of Six Common Skin Diseases Based on Xiangya-Derm: Development of a Chinese Database for Artificial Intelligence. *Journal of Medical Internet Research* 2021; **23**(9): e26025.
27. Ito H, Nakamura Y, Takanari K, et al. Development of a Novel Scar Screening System with Machine Learning. *Plastic and Reconstructive Surgery* 2022; **150**(2): 465e-72e.
28. Jain A, Way D, Gupta V, et al. Development and assessment of an artificial intelligence–based tool for skin condition diagnosis by primary care physicians and nurse practitioners in teledermatology practices. *JAMA network open* 2021; **4**(4): e217249-e.
29. Junayed MS, Islam MB, Jeny AA, Sadeghzadeh A, Biswas T, Shah AS. ScarNet: development and validation of a novel deep CNN model for acne scar classification with a new dataset. *IEEE Access* 2021; **10**: 1245-58.
30. Kim YJ, Han SS, Yang HJ, Chang SE. Prospective, comparative evaluation of a deep neural network and dermoscopy in the diagnosis of onychomycosis. *PLoS One* 2020; **15**(6): e0234334.

31. Lara JVM, Velásquez RMA. Low-cost image analysis with convolutional neural network for herpes zoster. *Biomedical Signal Processing and Control* 2022; **71**: 103250.
32. Lim ZV, Akram F, Ngo CP, et al. Automated grading of acne vulgaris by deep learning with convolutional neural networks. *Skin Research and Technology* 2020; **26**(2): 187-92.
33. Lin Y, Jiang J, Ma Z, et al. KIEGLFN: A unified acne grading framework on face images. *Computer Methods and Programs in Biomedicine* 2022; **221**: 106911.
34. Liu S, Fan Y, Duan M, et al. AcneGrader: An ensemble pruning of the deep learning base models to grade acne. *Skin Research and Technology* 2022; **28**(5): 677-88.
35. Liu Y, Jain A, Eng C, et al. A deep learning system for differential diagnosis of skin diseases. *Nature medicine* 2020; **26**(6): 900-8.
36. Luo W, Liu J, Huang Y, Zhao N. An effective vitiligo intelligent classification system. *Journal of Ambient Intelligence and Humanized Computing* 2020: 1-10.
37. Mathur J, Chouhan V, Pangti R, Kumar S, Gupta S. A convolutional neural network architecture for the recognition of cutaneous manifestations of COVID-19. *Dermatologic Therapy* 2021; **34**(2): e14902.
38. Melbin K, Jacob Vetha Raj Y. Automated detection and classification of skin diseases using diverse features and improved gray wolf-based multiple-layer perceptron neural network. *International Journal of Imaging Systems and Technology* 2021; **31**(3): 1317-33.
39. Muhaba KA, Dese K, Aga TM, Zewdu FT, Simegn GL. Automatic skin disease diagnosis using deep learning from clinical image and patient information. *Skin Health and Disease* 2022; **2**(1): e81.
40. Muñoz-López C, Ramírez-Cornejo C, Marchetti M, et al. Performance of a deep neural network in teledermatology: a single-centre prospective diagnostic study. *Journal of the European Academy of Dermatology and Venereology* 2021; **35**(2): 546-53.
41. Pangti R, Mathur J, Chouhan V, et al. A machine learning-based, decision support, mobile phone application for diagnosis of common dermatological diseases. *Journal of the European Academy of Dermatology and Venereology* 2021; **35**(2): 536-45.
42. Parekh R. Using texture analysis for medical diagnosis. *IEEE MultiMedia* 2012; **19**(2): 28.
43. Patil S, Rao ND, Patil A, Basar F, Bate S. Assessment of tibot® artificial intelligence application in prediction of diagnosis in dermatological conditions: results of a single centre study. *Indian Dermatology Online Journal* 2020; **11**(6): 910.
44. Saleh RE, Chantaf S, Naït-Ali A. Identification of facial skin diseases from face phenotypes using FSDNet in uncontrolled environment. *Machine Vision and Applications* 2022; **33**(2): 22.
45. Schaap M, Cardozo N, Patel A, De Jong E, Van Ginneken B, Seyger M. Image-based automated Psoriasis Area Severity Index scoring by Convolutional Neural Networks. *Journal of the European Academy of Dermatology and Venereology* 2022; **36**(1): 68-75.
46. Seité S, Khammari A, Benzaquen M, Moyal D, Dréno B. Development and accuracy of an artificial intelligence algorithm for acne grading from smartphone photographs. *Experimental dermatology* 2019; **28**(11): 1252-7.
47. Shanthi T, Sabeenian R, Anand R. Automatic diagnosis of skin diseases using convolution neural network. *Microprocessors and Microsystems* 2020; **76**: 103074.
48. Shen X, Zhang J, Yan C, Zhou H. An automatic diagnosis method of facial acne vulgaris based on convolutional neural network. *Scientific reports* 2018; **8**(1): 1-10.
49. Shrivastava VK, Londhe ND, Sonawane RS, Suri JS. A novel and robust Bayesian approach for segmentation of psoriasis lesions and its risk stratification. *Computer methods and programs in biomedicine* 2017; **150**: 9-22.
50. Thomsen K, Christensen AL, Iversen L, Lomholt HB, Winther O. Deep learning for diagnostic binary classification of multiple-lesion skin diseases. *Frontiers in medicine* 2020; **7**: 574329.
51. Verma S, Razzaque MA, Sangtongdee U, Arpnikanondt C, Tassaneetrithep B, Hossain A. Digital diagnosis of Hand, Foot, and mouth disease using hybrid deep neural networks. *IEEE Access* 2021; **9**: 143481-94.
52. Wen H, Yu W, Wu Y, et al. Acne detection and severity evaluation with interpretable convolutional neural network models. *Technology and Health Care* 2022; **30**(S1): 143-53.
53. Wu H, Yin H, Chen H, et al. A deep learning, image based approach for automated diagnosis for inflammatory skin diseases. *Annals of translational medicine* 2020; **8**(9).
54. Wu Z, Zhao S, Peng Y, et al. Studies on different CNN algorithms for face skin disease classification based on clinical images. *IEEE Access* 2019; **7**: 66505-11.
55. Yadav N, Alfayeed SM, Khamparia A, Pandey B, Thanh DN, Pande S. HSV model-based segmentation driven facial acne detection using deep learning. *Expert Systems* 2022; **39**(3): e12760.
56. Yan J, Liu F, Wang W. Scalable skin lesion multi-classification recognition system. *Computers, Materials & Continua* 2020; **62**(2): 801-16.
57. Yang Y, Guo L, Wu Q, et al. Construction and evaluation of a deep learning model for assessing acne vulgaris using clinical images. *Dermatology and Therapy* 2021; **11**(4): 1239-48.
58. Yang Y, Wang J, Xie F, et al. A convolutional neural network trained with dermoscopic images of psoriasis performed on par with 230 dermatologists. *Computers in Biology and Medicine* 2021; **139**: 104924.
59. Zhang L, Mishra S, Zhang T, et al. Design and assessment of convolutional neural network based methods for vitiligo diagnosis. *Frontiers in Medicine* 2021; **8**: 754202.
60. Zhang X, Wang S, Liu J, Tao C. Towards improving diagnosis of skin diseases by combining deep neural network and human knowledge. *BMC medical informatics and decision making* 2018; **18**(2): 69-76.
61. Zhao S, Xie B, Li Y, et al. Smart identification of psoriasis by images using convolutional neural networks: a case study in China. *Journal of the European Academy of Dermatology and Venereology* 2020; **34**(3): 518-24.

62. Zhao Z, Wu C-M, Zhang S, et al. A novel convolutional neural network for the diagnosis and classification of rosacea: usability study. *JMIR medical informatics* 2021; **9**(3): e23415.
63. Zhu C-Y, Wang Y-K, Chen H-P, et al. A deep learning based framework for diagnosing multiple skin diseases in a clinical environment. *Frontiers in medicine* 2021; **8**: 626369.
64. Zhu X, Zheng B, Cai W, et al. Deep learning-based diagnosis models for onychomycosis in dermoscopy. *Mycoses* 2022; **65**(4): 466-72.
